# A compilation of antimicrobial susceptibility data from a network of 13 Lebanese hospitals reflecting the national situation during 2015–2016

**DOI:** 10.1186/s13756-019-0487-5

**Published:** 2019-02-20

**Authors:** Rima Moghnieh, Georges F. Araj, Lyn Awad, Ziad Daoud, Jacques E. Mokhbat, Tamima Jisr, Dania Abdallah, Nadim Azar, Noha Irani-Hakimeh, Maher M. Balkis, Mona Youssef, Gilbert Karayakoupoglou, Monzer Hamze, Madonna Matar, Roula Atoui, Edmond Abboud, Rita Feghali, Nadine Yared, Rola Husni

**Affiliations:** 1 Department of Internal Medicine, Division of Infectious Diseases, Makassed General Hospital, Beirut, Lebanon; 2 Faculty of Medicine, Lebanese University, Beirut, Lebanon; 3 Professor and Director of Clinical Microbiology, Department of Pathology and Laboratory Medicine, American University of Beirut Medical Center, Beirut, Lebanon; 4 Pharmacy Department, Makassed General Hospital, Beirut, Lebanon; 5 Department of Microbiology, Centre Hospitalier du Nord, Zgharta, Lebanon; 6 Faculty of Medicine and Medical Sciences, University of Balamand, Koura, Lebanon; 7 Department of Internal Medicine, Division of Infectious Diseases, Lebanese American University-Rizk Hospital, Beirut, Lebanon; 8 Faculty of Medicine, Lebanese American University, Beirut, Lebanon; 9 Department of Laboratory Medicine, Makassed General Hospital, Beirut, Lebanon; 10 Department of Microbiology, Hotel Dieu de France Hospital, Beirut, Lebanon; 11 Department of Laboratory Medicine, Saint George University Hospital, Beirut, Lebanon; 12 Department of Internal Medicine, Division of Infectious Diseases, Labib Medical Center, Saida, Lebanon; 13 Medical Subspecialties Institute, Infectious Diseases, Cleveland Clinic Abu Dhabi, Abu Dhabi, United Arab Emirates; 14 Department of Internal Medicine, Division of Infectious Diseases, Haykel Hospital, Tripoli, Lebanon; 15 Department of Microbiology and Molecular diagnostics, Haykel Hospital, Tripoli, Lebanon; 16 Department of Microbiology, Nini Hospital, Tripoli, Lebanon; 17 Department of Internal Medicine, Division of Infectious Diseases, Notre Dame de Secours University Hospital, Jbeil, Lebanon; 18 Faculty of Medical Sciences, Holy Spirit University of Kaslik, Jounieh, Lebanon; 19 Department of Internal Medicine, Zahraa Hospital, Beirut, Lebanon; 20 Department of Microbiology, The Middle East Institute of Health University Hospital, Mount Lebanon, Lebanon; 21 Department of Laboratory Medicine, Rafik Hariri University Hospital, Beirut, Lebanon; 22 Chairperson Internal Medicine, Lebanese University, Beirut, Lebanon

**Keywords:** Antimicrobial susceptibility, Antimicrobial susceptibility testing, Resistance, Surveillance, Lebanon

## Abstract

**Background:**

There is a lack of official national antimicrobial resistance (AMR) data in Lebanon. Individual hospitals generate their own antibiotic susceptibility data in the form of yearly pamphlets.

**Methods:**

In this study, antibiotic susceptibility data from 13 hospitals distributed across different governorates of Lebanon were collected to conduct a compilation-based surveillance of AMR in Lebanon for the years 2015–2016. The findings were compared with those of a previous nationwide study in this country conducted between 2011 and 2013 as well as with similar data obtained from the 2015 and 2016 European surveillance reports of AMR. To provide a clear presentation of the AMR situation, mean percent susceptibility of different antibiotic–microbe combinations was calculated.

**Results:**

During 2015–2016, the percent susceptibility of *Enterobacteriaceae* to third-generation cephalosporins and to carbapenems was 59 and 97%, respectively. Among *Pseudomonas aeruginosa* and *Acinetobacter* spp., carbapenem susceptibility reached 70 and 12%, respectively. Among Gram positive organisms, the percent susceptibility to methicillin in *Staphylococcus aureus* was 72%, that to vancomycin in *Enterococcus* spp. was 98% and that to penicillin in *Streptococcus pneumoniae* was 75%. Compared with results of 2011–2013, there was an overall trend of decreased susceptibility of bacteria to the tested antibiotics, with a variation of 5 to 10%. The antibiotic susceptibility data from Lebanon were found to be comparable with those from Eastern and South-eastern European countries.

**Conclusion:**

This study highlights the need to establish a robust national AMR surveillance system that enables data from Lebanon to be included in global AMR maps.

**Electronic supplementary material:**

The online version of this article (10.1186/s13756-019-0487-5) contains supplementary material, which is available to authorized users.

## Background

Antimicrobial resistance (AMR) is a serious global public health concern. Recently, the prevalence of pandrug-resistant organisms has been reported with increasing frequency [1]. The World Health Organization (WHO) data on AMR have indicated increasing resistance in the Middle East and North Africa region [2]. A recent review on AMR prevalence in different countries of the Arab world has equally demonstrated increasing rates of resistance among multi-drug and extensively drug-resistant bacteria [3]. Although the Lebanese national AMR data are not well described in literature, Lebanon follows the same global trend of increasing AMR as reported by individual hospital studies [4–9]. In the absence of a standardised national surveillance in Lebanon, relatively large Lebanese hospitals generate yearly reports on their antibiotic susceptibility results of the corresponding year. These reports are distributed in the form of pamphlets to healthcare professionals and epidemiologists. Since 2017, workshops organised by the Lebanese Ministry of Health in collaboration with the WHO have been undertaken to evaluate work and improve the quality and capacity of microbiology laboratories in different regions of the country (unpublished data).

In 2015, a previous compilation of antibiotic susceptibility data of 16 Lebanese hospitals between the years 2011 and 2013 was published [10]. The present study is another follow-up attempt to generate antibiotic susceptibility data from a network of 13 Lebanese hospitals located in different regions of the country and to reflect on the current AMR situation for the years 2015 and 2016. Herein, we primarily addressed the evolution of antibiotic susceptibility by comparing the current results with those previously published [10]. In addition, we reviewed the methodologies of the participating hospital laboratories in reporting certain resistance patterns, aiming to include only those having the methodologies of identification and susceptibility testing of clinical isolates in line with international standards. A secondary aim was to compare this recent Lebanese data to similar ones from the European AMR Surveillance Network (EARS-Net) [11, 12], which is an already established AMR surveillance system of geographical proximity. The results of this study would ultimately raise awareness about the type of resistant organisms that should be a prioritised by AMR containment programmes in Lebanon. In addition, a platform can be laid for monitoring future AMR evolution, thereby aiding in future liaison with international AMR surveillance programmes.

## Methods

This is a retrospective study based on institutional antimicrobial susceptibility testing (AST) yearly reports/pamphlets generated and distributed by clinical microbiology laboratories of 13 hospitals located in different regions in Lebanon between 2015 and 2016. The institutional review board (IRB) Committee of one of the participating hospitals, Makassed General Hospital, Beirut, Lebanon, granted this study ethical approval. All data were purely based on microorganisms, and no patient data were included; hence, the IRB waived the requirement of informed consent from patients.

**Fig. 1:**
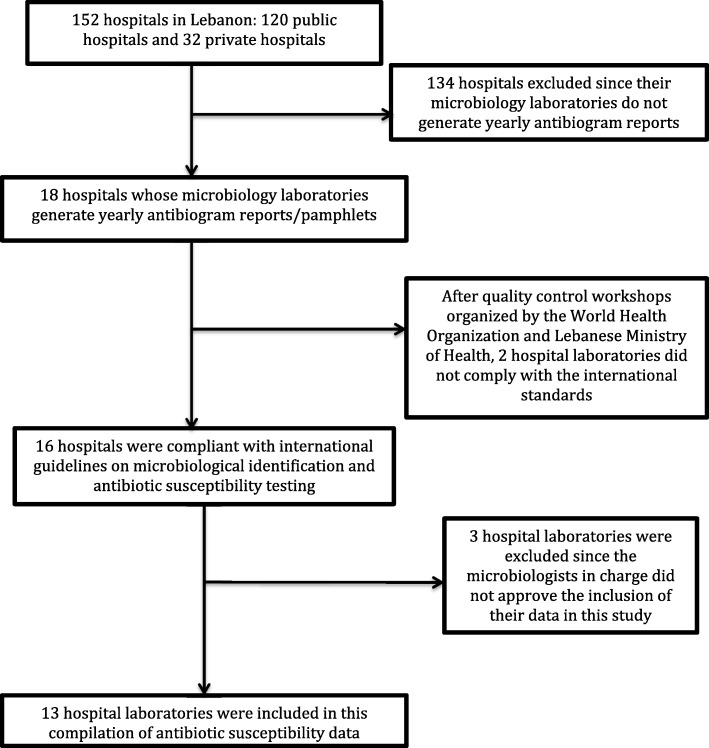
Flow diagram outlining the selection process for Lebanese hospital laboratories whose antibiotic susceptibility data was included in this study

Lebanon has 152 hospitals, 120 public and 32 private. We selected only 18 hospitals whose microbiology laboratories generated yearly antibiogram reports/pamphlets (Fig. 1). Two laboratories out of 18 were excluded since they did not comply with international microbiology guidelines [the Clinical & Laboratory Standards Institute (CLSI) and the European Committee on Antimicrobial Susceptibility Testing (EUCAST)] [13–17] in terms of identification and antibiotic susceptibility and/or their work was judged to be inadequate during quality control workshops undertaken by the Lebanese Ministry of Health and WHO in Lebanon. Although the antibiogram pamphlets were publicly available to healthcare professionals and epidemiologists, consent of the chief microbiologists of the chosen hospital laboratories was requested. Three microbiologists out of the remaining 16 did not approve the inclusion of their hospitals’ data in this study. Finally, antibiotic susceptibility data from 13 hospital laboratories were included (Fig. 1).

Bacterial isolates included in this study were recovered from different clinical specimens, e.g. urine, sputum, deep tracheal aspirates, blood, body fluids and the tips of medical devices. They represent a compilation of laboratory specimens originating from different patient populations including paediatric, adult, critically ill, pregnant patients, outpatients, etc. and they may have represented community-acquired or hospital-acquired infections. Identification and AST of bacteria were conducted according to the adopted methodology and interpretative standards of each laboratory.

Different aspects of AST were addressed with the participating hospital laboratories, including asking the chief microbiologist to specify additional tests conducted and surrogate markers used for detecting and confirming susceptibility or resistance of some pathogens to specific antimicrobial agents. These included: susceptibility of *Enterobacteriaceae* spp. to carbapenem, production of carbapenemase and extended-spectrum beta-lactamase (ESBL) in *Enterobacteriaceae* spp., susceptibility of *Salmonella* spp. to fluoroquinolone, susceptibility of *Streptococcus pneumoniae* to penicillin, methicillin resistance in *Staphylococcus aureus*, vancomycin resistance in *Enterococci*, susceptibility of *Pseudomonas aeruginosa* to colistin and susceptibility of *Acinetobacter* spp. to colistin (Additional file 1).

Data on specific organisms from any eligible laboratory that did not conform to the international guidelines [13–17] regarding the above additional questions was excluded from subanalysis.

Herein, we reported mean percent susceptibility of specific bacteria to individual antibiotics from the 13 hospitals according to a specific formula (Additional file 2). Besides presenting data as mean percent susceptibility for each antibiotic–microbe combination, we added a range of the upper and lower limits of the corresponding individual percent susceptibility from the included laboratories. When the number of isolates of an organism tested for susceptibility to a certain antibiotic was less than 20 per hospital, the corresponding AST result was excluded from calculations and subanalysis [18].

The percent susceptibility of *Enterobacteriaceae* spp. to antibiotics was represented by susceptibilities of *Escherichia coli* and *Klebsiella* spp. Susceptibility of Gram negative bacteria to ceftriaxone represented susceptibility to third-generation cephalosporins (3GC), except in the case of *P. aeruginosa* where susceptibility to ceftazidime was studied. Imipenem, unlike other carbapenems, was consistently used in all hospitals, and hence, it was considered as a marker of carbapenem susceptibility in different Gram negative species in this study. If the carbapenemase or ESBL production screening was positive, further confirmatory tests were required to establish the presence of these resistance mechanisms [13, 14, 17]. Accordingly, data from laboratories that conformed to the guidelines concerning this issue were included for calculating the mean percentage of ESBL and carbapenemase production in *Enterobacteriaceae*. This value did not depend on antibiogram results alone. Regarding *S. pneumoniae*, we excluded data from laboratories that determined susceptibility to penicillin based only on the oxacillin DD method, as this might have been an overestimation of penicillin non-susceptibility (Additional file 1).

The 2015–2016 results were compared with those from an earlier nationwide study, including compiled susceptibility data from 16 Lebanese hospitals between 2011 and 2013 [10]. Ten out of the 13 included hospitals in the current study participated in the previous one.

We also compared the 2015–2016 Lebanese results with similar results from the 2015 and 2016 EARS-Net reports of AMR [11, 12]. For the 2015 and 2016 European data, the mean percent susceptibility was calculated using a specific formula (Additional file 2).

### Statistical analysis

The 2015–2016 and the 2011–2013% antibiotic susceptibility results were compared using odd ratios (ORs) and chi-squared *p*-values calculated using the OR function of the epitools package in the R statistical program [19]. ORs were computed with the 2011–2013% antibiotic susceptibility data set as a reference. OR < 1 indicates a decrease in percent antibiotic susceptibility in 2015–2016 vs. 2011–2013. OR > 1 indicates an increase in percent antibiotic susceptibility in 2015–2016 vs. 2011–2013. The 2015–2016 Lebanese data were compared with the 2015–2016 European data in the same manner using the European results as a reference. OR < 1 indicates a decreased percent susceptibility while OR > 1 indicates an increased percent susceptibility in Lebanon compared to the European countries. The Bonferroni method was used for adjusting the *p*-value for multiple testing. A p-value of < 0.05 was considered to be statistically significant.

## Results

### Microbiological techniques and AST methods

The included hospitals represented all geographical zones/governorates of the country, except for the Bekaa Valley where data were unavailable and no laboratory could fulfil our inclusion criteria. They encompassed 10 university and 3 community hospitals. AST techniques used included automation in 5 hospitals, manual methods in 7 hospitals and both in 1 hospital. The antibiotic susceptibility interpretive standards were based on the CLSI guidelines in 9 hospitals, the EUCAST guidelines in 2 hospitals and both in 2 hospitals (Table 1).

**Table 1:** Demographics and antibiotic susceptibility testing methods in the 13 participating hospitals^a^

Hospital	Region	Type	Beds	Microbiological Method^b^	Guidelines
HDF	Beirut	University	444	Automated	EUCAST
LAUMC-RH	Beirut	University	114	Manual	CLSI + EUCAST
RHUH	Beirut	University	250	Automated	CLSI
AUBMC	Beirut	University	380	Manual	CLSI
MGH	Beirut	University	170	Manual	CLSI
Zahraa	Mount Lebanon	University	201	Manual + Automated	CLSI
CHU-NDS	Mount Lebanon	University	250	Automated	CLSI + EUCAST
MEIH	Mount Lebanon	University	150	Manual	EUCAST
SGH	Mount Lebanon	University	400	Manual	CLSI
Nini	North Lebanon	Community	175	Manual	CLSI
CHdN	North Lebanon	University	180	Manual	CLSI
Haykal	North Lebanon	Community	120	Automated	CLSI
Labib MC	South Lebanon	Community	120	Automated	CLSI

KEY: *HDF* Hotel Dieu de France, *LAUMC-RH* Lebanese American University Medical Center – Rizk Hospital, *RHUH* Rafic Hariri University Hospital, *AUBMC* American University of Beirut Medical Center, *MGH* Makassed General Hospital, *CHU-NDS* Centre Hospitaler Universitaire Notre Dame de Secours, *MEIH* Middle East Institute of Health, *SGH* Saint Georges Hospital, *CHdN* Centre Hospitalier du Nord, *MC* Medical Center, *EUCAST* European Committee on Antimicrobial Susceptibility Testing, *CLSI* Clinical and Laboratory Standards Institute

^a^All of the participating hospitals are private, except for RHUH that is a public hospital

^b^Automated microbial identification system: Vitek, BD Phoenix

The microbiological methods used in the included laboratories were compliant with the CLSI and EUCAST standards. However, we observed deviation from international guidelines with regard to susceptibility testing for certain antibiotic-microbe combinations, such as susceptibilities of *Acinetobacter* spp. to colistin, *S. pneumoniae* to penicillin and *Salmonella* spp. to fluoroquinolone. In addition, *Enterococcus faecium* and *E. faecalis* were grouped together in majority of the hospitals reports and corresponding antibiotic susceptibility patterns were reported for the *Enterococcus* genus without differentiation between the two species. As for *Acinetobacter* spp. susceptibility to colistin, all laboratories did not use the broth microdilution method, which is recommended by CLSI and EUCAST guidelines. Instead, 6/13 laboratories used automated minimal inhibitory concentration (MIC) method, 5/13 laboratories used disc diffusion (DD) method, and 2/13 laboratories did not report results (Additional file 1). So, we did not report colistin susceptibility results in *Acinetobacter* spp. herein.

Upon comparing the methods in this study to that of 2011–2013 study, i.e. in terms of the source of data and the calculation of percent susceptibility, both studies were comparable. Concerning the methods used in detecting and confirming susceptibility or resistance of some pathogens to specific antimicrobial agents that we mentioned earlier, both studies were comparable as well, except in the detection of ESBL and carbapenemase production in *Enterobacteriaceae* (personal communication) (Additional file 1). However, comparison was made only between 3GC and carbapenem susceptibility results in *Enterobacteriaceae* and that related to detection of ESBL or carbapeneamase enzymes was not carried on between the 2 study periods.

### Types of bacteria and antibiotic susceptibility results

**Fig. 2:**
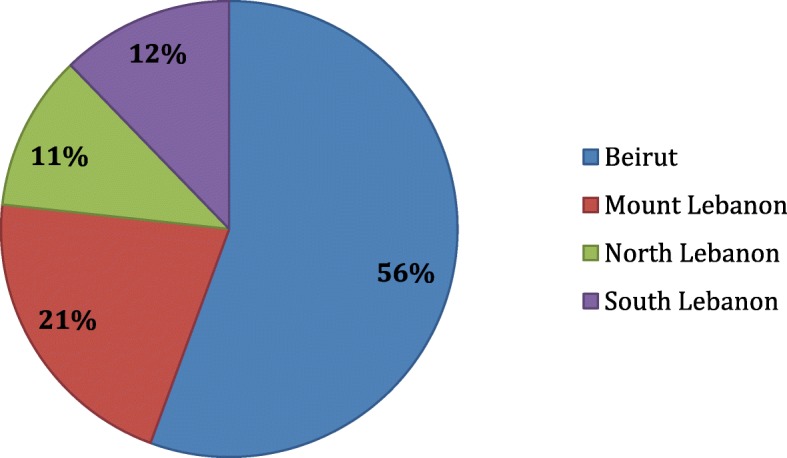
Distribution of the total clinical isolates in 2015 and 2016 (%) among the governorates of Lebanon (*N* = 85,144 isolates)

During the study period of 2015–2016, a total of 85,144 clinical isolates were included in this compilation antibiogram: 76% were Gram negative and 24% were Gram positive organisms. The distribution of the tested isolates per governorate is depicted in Fig. 2.

Among the tested *Enterobacteriaceae* spp., the mean percent susceptibility to antibiotics was as follows: 59% to 3GC (58% in *E. coli* and 63% in *Klebsiella* spp.), 77% to cefoxitin (76% in *E. coli* and 83% in *Klebsiella* spp.), 97% to carbapenems (97% in *E. coli* and 96% in *Klebsiella* spp.), 60% to ciprofloxacin (57% in *E. coli* and 71% in *Klebsiella* spp.), 91% to amikacin (90% in *E. coli* and 94% in *Klebsiella* spp.), 54% to trimethoprim-sulfamethoxazole (53% in *E. coli* and 58% in *Klebsiella* spp.) and 83% to nitrofurantoin (87% in *E. coli* and 59% in *Klebsiella* spp.). (Table 2).

**Table 2:** Percent antibiotic susceptibility of *E. coli*, and *Klebsiella* spp. during 2015/2016 compared to corresponding susceptibility results in 2011/2013^a^

Antibiotic	*E. coli*				*Klebsiella* spp			
	2011/2013 *N* (%S)	2015/2016 *N* (%S)	OR (95%CI)	*P*	2011/2013 *N* (%S)	2015/2016 *N* (%S)	OR (95% CI)	*P*
Amikacin	29,667 (97%)	41,818 (90%)	0.29 (0.26–0.3)	0.0001	7768 (96%)	9498 (94%)	0.65 (0.57–0.75)	0.0001
Amoxicillin/clavulanic acid	30,411 (61%)	39,116 (59%)	1.13 (1.10–1.17)	0.0001	7883 (67%)	9069 (62%)	0.80 (0.75–0.86)	0.0001
Ampicillin	22,985 (23%)	35,906 (24%)	1.06 (1.02–1.10)	0.01	–	–	–	–
Aztreonam	27,221 (66%)	40,029 (57%)	0.65 (0.63–0.67)	0.0001	7020 (69%)	7924 (65%)	0.83 (0.78–0.90)	0.0001
Cefepime	26,314 (74%)	40,524 (62%)	0.60 (0.58–0.62)	0.0001	–	–	–	–
Cefoxitin	23,858 (87%)	33,104 (76%)	0.47 (0.45–0.50)	0.0001	5853 (89%)	6373 (83%)	0.60 (0.54–0.67)	0.0001
Ceftazidime	28,730 (71%)	41,816 (62%)	0.73 (0.71–0.76)	0.0001	7335 (71%)	9498 (64%)	0.73 (0.68–0.78)	0.0001
Ceftriaxone	20,059 (64%)	41,816 (58%)	0.85 (0.82–0.88)	0.0001	4750 (65%)	9498 (63%)	0.92 (0.85–0.99)	0.02
Cefuroxime	24,662 (59%)	33,106 (56%)	0.96 (0.93–0.99)	0.02	6516 (64%)	6579 (61%)	0.88 (0.82–0.94)	0.0004
Ciprofloxacin	29,411 (55%)	40,524 (57%)	1.22 (1.19–1.26)	0.0001	7883 (73%)	9311 (71%)	0.91 (0.85–0.97)	0.004
Gentamicin	29,327 (72%)	41,818 (72%)	1.00 (0.98–1.03)	1	7601 (75%)	9498 (78%)	1.18 (1.10–1.27)	0.0001
Imipenem	30,411 (99%)	41,813 (97%)	0.33 (0.29–0.37)	0.0001	7883 (98%)	9498 (96%)	0.49 (0.41–0.60)	0.0001
Nitrofurantoin	18,422 (96%)	29,434 (87%)	0.28 (0.26–0.30)	0.0001	4356 (52%)	5155 (59%)	1.33 (1.22–1.44)	0.0001
Piperacillin/tazobactam	28,739 (83%)	40,524 (76%)	0.84 (0.81–0.873)	0.0001	7618 (81%)	9292 (78%)	0.83 (0.77–0.90)	0.0001
Tigecycline	9716 (98%)	39,050 (96%)	0.24 (0.20–0.30)	0.0001	2243 (87%)	8546 (93%)	1.99 (1.71–2.31)	0.0001
Trimethoprim/sulfamethoxazole	29,689 (49%)	41,496 (53%)	1.13 (1.10–1.16)	0.0001	7709 (57%)	9498 (58%)	1.04 (0.98–1.11)	0.2

KEY = *CI* confidence interval, ***N*** Number of isolates tested in each bacteria/antibiotic combination, *OR* Odds Ratio, *S* Susceptibility, *%* percent

*P* < 0.05 is considered statistically significant

^a^The 2011/2013 data are taken from: Chamoun K, Farah M, Araj G, et al. Surveillance of antimicrobial resistance in Lebanese hospitals: retrospective nationwide compiled data. Int J Infect Dis. 2016;46:64–70

The proportion of ESBL and carbapenemase production among *Enterobacteriaceae* did not coincide with the susceptibility results obtained for 3GC and carbapenems, respectively, because for reporting the presence of these enzymes, we selectively included those laboratories that reported their findings based on confirmatory tests used to detect the presence of these resistance mechanisms and did not just depend on the antibiogram results. Detection of ESBL production in *Enterobacteriaceae* spp. was reported by 8 out of 13 hospitals and carbapenemase production by 2 out of 13 hospitals. ESBL production was observed in 34% of *Enterobacteriaceae* spp. (ranging between 25 and 43%) from 8 of the 13 laboratories that followed the international guidelines for detecting this resistance mechanism. Similarly, the mean proportion of carbapenemase production was 0.6% in case of *E. coli* and 2% in case of *Klebsiella* spp. as generated from only 2 of the 13 laboratories (Additional file 3).

The susceptibility results of *P*. *aeruginosa* isolates from all hospitals were as follows: 80% to ceftazidime, 78% to piperacillin/tazobactam, 70% to imipenem, 69% to meropenem, 73% to ciprofloxacin, 85% to amikacin and 98% to colistin (Table 3). Percent susceptibility of *P. aeruginosa* to carbapenems among all centres ranged from 55 to 95% (Additional file 4).

**Table 3:** Percent antibiotic susceptibility of *Acinetobacter* spp. and *P. aeruginosa* during 2015/2016 compared to corresponding susceptibility results in 2011/2013^a^

Antibiotic	*Acinetobacter* spp				*P. aeruginosa*			
	2011/2013 *N* (% S)	2015/2016 *N* (% S)	OR (95% CI)	*P*	2011/2013 *N* (% S)	2015/2016 *N* (% S)	OR (95% CI)	*P*
Amikacin	3329 (16%)	3675 (19%)	1.23 (1.11–1.36)	0.0001	7675 (89%)	9005 (85%)	0.70 (0.62–0.79)	0.0001
Aztreonam	NA	NA	NA	NA	7483 (76%)	7457 (79%)	1.19 (1.06–1.33)	0.002
Cefepime	3409 (13%)	3675 (13%)	1.00 (0.89–1.12)	1	7897 (83%)	9005 (81%)	0.87 (0.79–0.97)	0.01
Ceftazidime	3343 (12%)	3675 (13%)	1.10 (0.973–1.23)	0.14	7897 (82%)	9005 (80%)	0.88 (0.79–0.97)	0.01
Ciprofloxacin	3379 (12%)	3675 (11%)	0.91 (0.80–1.02)	0.12	7897 (77%)	9005 (73%)	0.81 (0.74–0.89)	0.0001
Gentamicin	3384 (19%)	3675 (14%)	0.70 (0.62–0.78)	0.0001	7722 (83%)	9005 (81%)	0.87 (0.79–0.97)	0.01
Imipenem	3409 (18%)	3675 (12%)	0.62 (0.56–0.70)	0.0001	7897 (73%)	9005 (70%)	0.86 (0.79–0.94)	0.001
Piperacillin/tazobactam	3343 (13%)	3675 (11%)	0.83 (0.73–0.93)	0.12	7897 (80%)	9005 (78%)	0.89 (0.80–0.98)	0.02

KEY = *CI* confidence interval, *N* Number of isolates tested in each bacteria/antibiotic combination, *NA* not available, *OR* Odds Ratio, *S* Susceptibility, *%* percent

*P* < 0.05 is considered statistically significant

^a^The 2011/2013 data are taken from: Chamoun K, Farah M, Araj G, et al. Surveillance of antimicrobial resistance in Lebanese hospitals: retrospective nationwide compiled data. Int J Infect Dis. 2016;46:64–70

Regarding *Acinetobacter* spp., mean percent antibiotic susceptibility was calculated from 11 hospitals where 12% of the tested isolates were susceptible to carbapenems ranging from 3 to 74% (Additional file 4) and 80% were susceptible to tigecycline (Table 3).

The antibiotic susceptibility of *Salmonella* spp. was tested in 10 hospitals, where 97% of the isolates were found to be susceptible to 3GC, 90% to ciprofloxacin and 71% to trimethoprim-sulfamethoxazole (Table 4). Herein, the range of fluoroquinolone susceptibility in *Salmonella* spp. among centres was from 56 to 100% (Additional file 5).

**Table 4:** Percent antibiotic susceptibility of *Salmonella* spp. during 2015/2016 compared to corresponding susceptibility results in 2011/2013^a^

Antibiotic	2011/2013 *N* (%S)	2015/2016 *N* (%S)	OR (95% CI)	*P*
Ampicillin	784 (81%)	596 (85%)	1.34 (1.00–1.79)	0.06
Ceftriaxone	690 (97%)	655 (97%)	0.99 (0.53–1.87)	1
Ciprofloxacin	877 (95%)	721 (90%)	0.48 (0.32–0.70)	0.0001
Trimethoprim/sulfamethoxazole	877 (92%)	721 (71%)	0.21 (0.16–0.28)	0.0001

KEY = *CI* confidence interval, *N* Number of isolates tested in each bacteria/antibiotic combination, *OR* Odds Ratio, *S* Susceptibility, ***%*** percent

*P* < 0.05 is considered statistically significant

^a^The 2011/2013 data are taken from: Chamoun K, Farah M, Araj G, et al. Surveillance of antimicrobial resistance in Lebanese hospitals: retrospective nationwide compiled data. Int J Infect Dis. 2016;46:64–70

Among *S. aureus* isolates, the susceptibility results were as follows: 72% to oxacillin/methicillin, 81% to ciprofloxacin, 81% to clindamycin and 73% to erythromycin (Table 5). Although one would expect all isolates to be completely susceptible to vancomycin, one laboratory reported a resistance of 4% using the automated system. Susceptibility to oxacillin/methicillin ranged from 52 to 86% among all centres (Additional file 6).

**Table 5:** Percent antibiotic susceptibility of gram-positive bacteria (*S. aureus, S. pneumoniae, Enterococcus spp*.) during 2015/2016 compared to corresponding susceptibility results in 2011/2013^a^

Antibiotic	*S. aureus*				*S. pneumoniae*				*Enterococcus spp.*			
	2011/2013 *N* (%S)	2015/2016 *N* (%S)	OR (95% CI)	*P*	2011/2013 *N* (%S)	2015/2016 *N* (%S)	OR (95% CI)	*P*	2011/2013 *N* (%S)	2015/2016 *N* (%S)	OR (95% CI)	*P*
Ampicillin	NA	NA	NA	NA	NA	NA	NA	NA	3847 (84%)	3760 (75%)	0.57 (0.51–0.64)	0.0001
Clindamycin	4359 (83%)	6452 (81%)	0.87 (0.79–0.96)	0.01	588 (76%)	584 (75%)	0.95 (0.73–1.24)	1	NA	NA	NA	NA
Erythromycin	4890 (76%)	6304 (73%)	0.85 (0.78–0.93)	0.0004	544 (63%)	584 (65%)	1.09 (0.86–1.39)	1	NA	NA	NA	NA
Levofloxacin	2297 (84%)	2873 (72%)	0.49 (0.43–0.56)	0.0001	483 (91%)	584 (99%)	1.07 (0.72–1.40)	1	NA	NA	NA	NA
Oxacillin	4752 (73%)	6452 (72%)	0.95 (0.87–1.03)	0.25	NA	NA	NA	NA	NA	NA	NA	NA
Tigecycline	492 (99%)	1788 (99%)	1.03 (0.33–2.63)	0.985	NA	NA	NA	NA	723 (99%)	1022 (99%)	0.10 (0.35–1.64)	0.983
Trimethoprim/sulfamethoxazole	4604 (91%)	6437 (88%)	0.73 (0.64–0.82)	0.0001	296 (53%)	398 (55%)	1.08 (0.80–1.47)	1	NA	NA	NA	NA
Vancomycin	4890 (100%)	6452 (100%)	1.32 (0.03–1.47)	0.819	NA	NA	NA	NA	4145 (99%)	3760 (98%)	0.49 (0.33–0.72)	0.0003

KEY = *CI* confidence interval, *N* Number of isolates tested in each bacteria/antibiotic combination, *NA* not available**,** *OR* Odds Ratio, *S* Susceptibility, % percent

*P* < 0.05 is considered statistically significant

^a^The 2011/2013 data are taken from: Chamoun K, Farah M, Araj G, et al. Surveillance of antimicrobial resistance in Lebanese hospitals: retrospective nationwide compiled data. Int J Infect Dis. 2016;46:64–70

Among isolated *Enterococci* spp., the susceptibility results were as follows: 75% to ampicillin, 98% to vancomycin and 99% to both tigecycline and linezolid (Table 5). Ampicillin and vancomycin susceptibility ranged from 46 to 96% and from 83 to 100% among all centres, respectively (Additional file 6).

Regarding *S*. *pneumoniae*, three hospitals were compliant with the CLSI and EUCAST guidelines and determined penicillin MIC. Of the tested isolates from three of the eight hospitals that reported *S. pneumoniae* susceptibility results, 75% were susceptible to penicillin, with the individual institutional susceptibility ranging between 60 and 79%. Mean fluoroquinolone susceptibility was 99%using data from 8 hospitals. The antibiotic susceptibility results for all *Streptococcus* spp. are shown in “Additional file 7”. One laboratory reported penicillin resistance in *S*. *agalactiae* (3%) using the automated system.

### Comparison with the 2011–2013 antibiotic susceptibility results

The 2011–2013 compilation of antibiotic susceptibility data has locally been considered as the most representative report on national AMR surveillance in Lebanon for that time period. When the current data were compared with those of 2011–2013, there were few results that were not mentioned in the former study, such as susceptibilities of *P. aeruginosa* to colistin, *Enterococcus* spp. to linezolid and *S. pneumoniae* to penicillin.

In general, we observed that the variation in the percent susceptibility in majority of the antibiotic–microbe combinations between both periods was less than 10%, primarily exhibiting a trend of decreasing susceptibility.

**Fig. 3:**
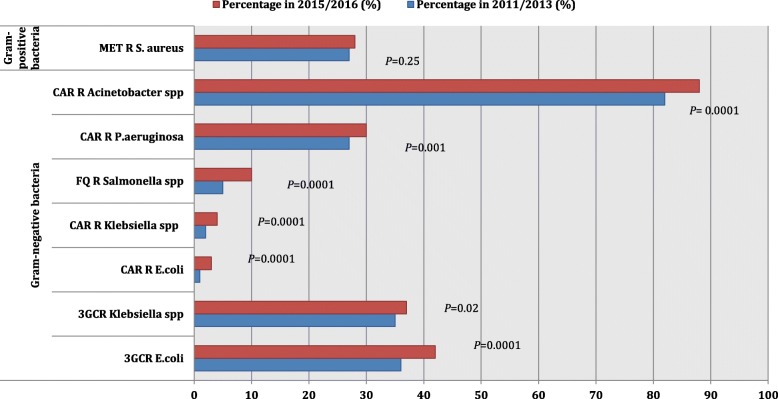
Percentage of resistance among six clinically important bacteria in Lebanon during 2011–2013 and 2015–2016, described by the World Health Organization as priority organisms for research and development of new antimicrobials. KEY: *CAR* carbapenem, *FQ* fluoroquinolone, *MET* methicillin, *3GC* third-generation cephalosporins, *R* resistant. N.B. *P* < 0.05 is considered statistically significant

A decreasing trend in antibiotic susceptibility (between 5 and 10%) was observed in the following cases: *E. coli* to nitrofurantoin, *Klebsiella* spp. to cefoxitin, *Acinetobacter* spp. to imipenem, *Salmonella* spp. to ciprofloxacin and *Enterococci* to ampicillin (Tables 2, 3, 4 and 5) (Fig. 3).

A decrease in antibiotic susceptibility (less than 5%) was observed in the following cases: *E. coli* to imipenem, *Klebsiella* spp. to imipenem, *P. aeruginosa* to imipenem and *S. aureus* to oxacillin/methicillin (Tables 2, 3 and 5) (Fig. 3).

However, there was a considerable decrease in antibiotic susceptibility (10% or more) in the following organisms: *E. coli* to cefepime, *E. coli* to cefoxitin and *Salmonella* spp. to trimethoprim-sulfamethoxazole and *S. aureus* isolates to levofloxacin (Tables 2, 4 and 5).

### Comparison with 2015–2016 EARS-net data

For the comparison of the 2015–2016 Lebanese data to European surveillance data during the same period, we focused on the following antibiotic-bacteria susceptibility patterns: *E.coli* and *Klebsiella* spp. percent susceptibility to 3GC and to carbapenems each separately, *P. aeruginosa* and *Acinetobacter* spp. percent susceptibility to carbapenems each separately, *S. aureus* percent susceptibility to methicillin, and *S. pneumoniae* percent susceptibility to penicillin.

Upon comparing the Lebanese and European findings, we noticed that the Lebanese percent antibiotic susceptibilities in the formerly stated organisms were lower than those reported from Northern and Western European countries and were similar or close to those in Eastern and South-eastern Europe.

**Fig. 4:**
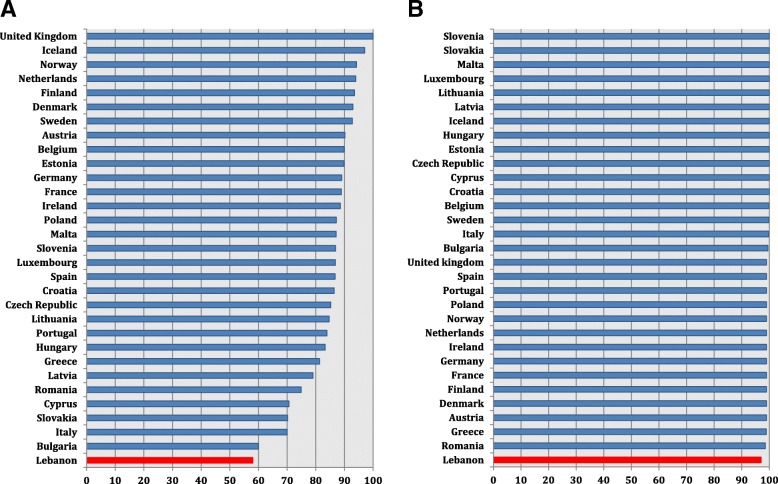
*E. coli* percent susceptibility to third-generation cephalosporins (**a**) and to carbapenems (**b**) in Lebanon in 2015 and 2016 in comparison with similar data from countries of the European Union, based on the 2015 and 2016 annual reports of the European Antimicrobial Resistance Surveillance Network (EARS-Net)

Regarding *E. coli*, Lebanon reported the lowest percent susceptibility to 3GC (58%) compared to findings from Italy (70.1%), Greece (81.3%), and Spain (86.7%) (Fig. 4) (Additional file 8). Carbapenem susceptibility in *E. coli* was also the lowest in Lebanon (97%) yet close to that from Romania (98.6%), and lower than values from Italy (99.8%) and Greece (98.95%) (Fig. 4) (Additional file 9).

**Fig. 5:**
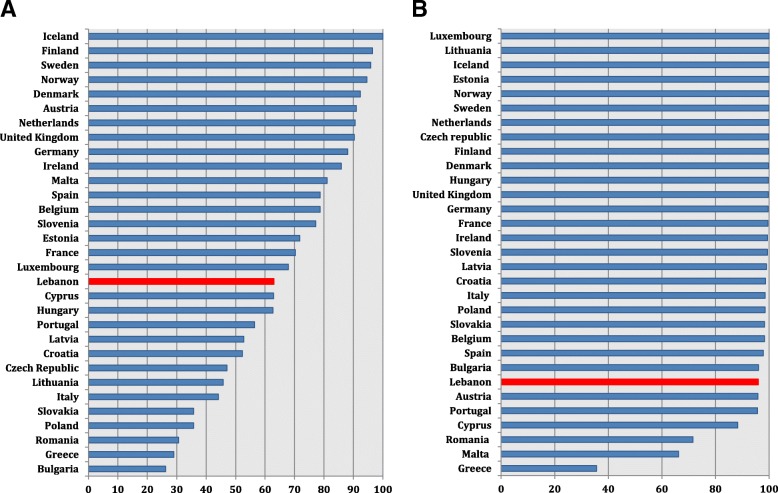
*K. pneumoniae* percent susceptibility to third-generation cephalosporins (**a**) and to carbapenems (**b**) in Lebanon in 2015 and 2016 in comparison with similar data from countries of the European Union, based on the 2015 and 2016 annual reports of the European Antimicrobial Resistance Surveillance Network (EARS-Net)

As for *Klebsiella* spp., 3GC and carbapenem percent susceptibility in Lebanon were not the lowest. 3GC susceptibility (63%) was similar to that reported from Luxembourg (67.9%), Hungary (62.7%), and Cyprus (62.9%). It was higher than that from Greece (29%) and Italy (44.2%), yet lower than Spain (78.7%) (Fig. 5) (Additional file 10). Regarding carbapenem susceptibility, our result (96%) was similar to that from Bulgaria (96.1%), higher than that from Greece (35.6%) and lower than that from Spain (97.9%) (Fig. 5) (Additional file 11).

**Fig. 6:**
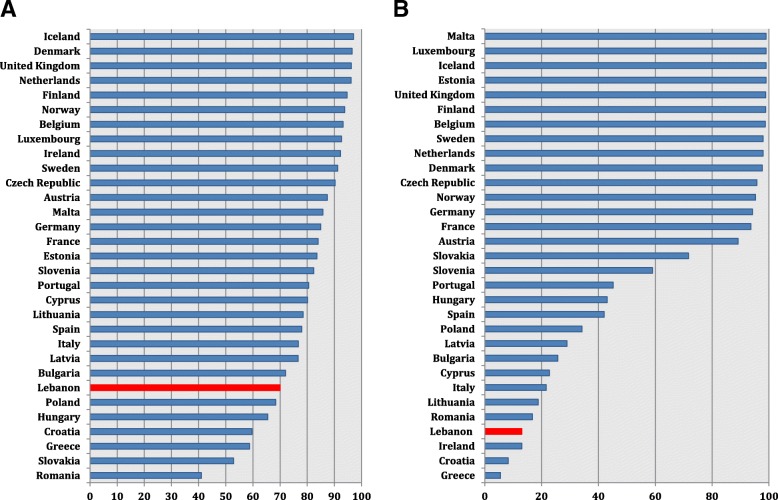
*P. aeruginosa* (**a**) and *Acinetobacter* spp. (**b**) percent susceptibility to carbapenems in Lebanon in 2015 and 2016 in comparison with similar data from countries of the European Union, based on the 2015 and 2016 annual reports of the European Antimicrobial Resistance Surveillance Network (EARS-Net)

As for carbapenem percent susceptibility in *P. aeruginosa*, Lebanon was among countries reporting a low level (70%) but was not the lowest. Susceptibility data reported from Poland (68.4%) and Bulgaria (72.1%) were similar to ours. Our carbapenem susceptibility was higher than that from Greece (58.8%) and Romania (41.1%), and lower than that from Italy (76.8%) and Spain (78%) (Fig. 6) (Additional file 12).

In Lebanon, carbepenem percent susceptibility in *Acinetobacter* spp. is very low (13%), yet our country shared similar results with Romania (16.8%) (Fig. 6) (Additional file 13). Values were lower from Croatia (8.3%) and Greece (5.6%), higher from Italy (21.6%), and much higher from Spain (42%).

**Fig. 7:**
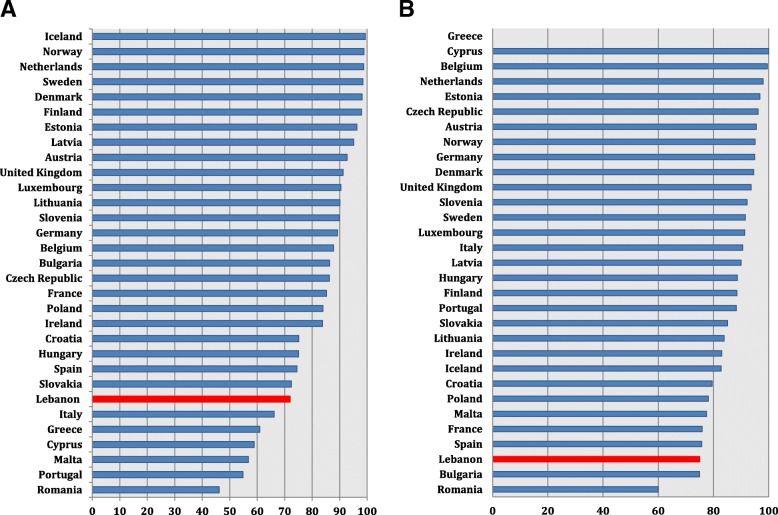
*S. aureus* percent susceptibility to methicillin (**a**) and *S. pneumonaie* percent susceptibility to penicillin (**b**) in Lebanon in comparison with countries of the European Union during 2015 and 2016, based on the 2015 and 2016 annual reports of the European Antimicrobial Resistance Surveillance Network (EARS-Net)

Concerning Gram positive organisms, Lebanese results on *S. aureus* susceptibility to methicillin (72%) were comparable with those from Slovakia (72.4%) and Spain (74.5%), and higher than findings from Italy (66.2%) and Greece (60.9%) (Fig. 7) (Additional file 14). Penicillin susceptibility in *S. pneumoniae* reached 75% in Lebanon. This value was similar to those reported from Bulgaria (74.9%), Spain (75.8%), and France (75.9%). It was lower than findings from Italy (90.6%). Data from Greece were not available (Fig. 7) (Additional file 15).

## Discussion

### Epidemiologic representativeness of the bacterial Pool

This study reports a compilation of antibiotic susceptibility data retrieved from the antibiogram pamphlets generated by 13 hospital laboratories located in different Lebanese governorates, whose work was standardised and compliant with the CLSI or the EUCAST guidelines on bacterial identification and AST [13–17]. Only 13 of the 150 hospital laboratories were included because the remaining hospitals did not fulfil the inclusion criteria. This highlights the need for improving the performance of microbiology laboratories through standardisation and external quality control. Since 2017, workshops, aiming at capacity building and improving the performance of microbiology laboratories nationwide, have been held by the Lebanese Ministry of Health and supported by the WHO (unpublished data).

In countries where official national or international AMR surveillance is not applied, the available antibiotic susceptibility data are considered to be representative of the countries [3]. In early 2018, the WHO released the first Global Antimicrobial Resistance Surveillance System (GLASS) report in its early implementation phase (2016–2017), which included AMR data from two Lebanese hospital laboratories whose 2015–2016 AMR data are included in our study [20]. The number of organisms included in this compilation (85,144) outweighs the number of organisms included in the GLASS data from Lebanon (1309), and in the latter, data collection was during 2016 only [20]. Data on *P. aeruginosa* susceptibility to different antibiotics was non-available in the GLASS report [20]. This situation emphasizes the necessity for establishing an official surveillance system that should be included in the national plan for controlling the emergence and spread of AMR.

### Range of antibiotic percent susceptibility among the Lebanese hospitals

In this study, we observed a wide range in the reported percent susceptibility among the tested organisms to certain antibiotics such as in the case of susceptibilities of *Klebsiella* spp. to 3GC (35–72%), *Acinetobacter* spp. to carbapenems (3–74%), *P. aeruginosa* to carbapenems (55 to 95%), and *S. aureus* to oxacillin (52–86%). These organisms are major causative agents of healthcare-associated infections. Accordingly, we hypothesized that variability in the reported resistance patterns in these organisms might be due to different resistant clones circulating in different hospitals [5, 6, 21, 22]. A multicentre nationwide cross-sectional study investigated the prevalence and molecular basis of carbapenem resistance in Gram-negative bacteria collected from 11 tertiary care hospitals, geographically distributed over 5 Lebanese Governorates in 2012 [6]. Two of these hospitals provided AST data in our study herein, one from Beirut and the other from South Lebanon. Investigators reported high-level carbapenem resistance in *A. baumannii* and *P. aeruginosa*, 88% (638/724 isolates) and 41% (760/1848 isolates), respectively [6]. Ninety percent of the carbapenem-resistant *A. baumanni* produced OXA-23 carbapenemase and 16% of the carbapenem-resistant *P. aeruginosa* isolates harboured VIM-2 metallo-b-lactamase. In addition, the diffusion of these enzymes was primarily due to clonal dissemination [6]. Another recent multicentre study by Atrouni et al. investigated the molecular epidemiology of *A. baumannii* strains isolated from various types of clinical specimens from 7 hospitals situated in different regions in Lebanon between 2013 and 2015 [5]. Investigators showed that out of 119 non-duplicate isolates, 76.5% were resistant to carbapenems and the most common carbapenemase was the OXA-23-type, found in 82 isolates [5]. In this study, 2 out of 7 centres were included in our current study, one from Beirut and the other from North Lebanon. The study by Atrouni et al. highlighted the nationwide dissemination of highly related OXA-23-producing carbapenem-resistant *A. baumannii* in Lebanese hospitals [5].

Regarding *Enterobacteriaceae*, 3GC susceptibility was not 100% in any of the included hospitals, where the upper limit of the range for susceptibility of 3GC was 70% for *E. coli* and 72% for *Klebsiella spp.* meaning that all reporting hospitals detected resistance to 3GC at different degrees (at least 30% in *E. coli* and 28% in *Klebsiella* spp). This finding could be explained by that 3GC resistance in *Enterobacteriaceae* has possibly spread in the community and reached the hospital setting from the community. As mentioned previously, the tested organisms in this study originated from pooled hospital laboratory data, where there was no differentiation between hospital-acquired or community-acquired infections. A single centre study from a university hospital in Beirut evaluating the epidemiology of bacteremia in cancer patients showed that the percentage of hospital-acquired 3GC resistance in *Enterobacteriaceae* causing bacteremia was 51% and that broad-spectrum antibiotic exposure during hospital stay was a risk factor for the acquisition of 3GC-resistant organisms [23]. In another Lebanese study on community-acquired urinary tract infections caused by *E. coli* and *Klebsiella* spp. collected from 3 hospitals, Moghnieh et al. found that the percentage of 3GC resistance was 31% in *E. coli* and 29% in *Klebsiella* spp. during 2010 and 2011 [24].

Similarly, the range of susceptibility of *Enterobacteriaceae* to fluoroquinolones was 44–74% in *E. coli* and 56–80% in *Klebsiella* spp. So *E. coli* and *Klebsiella* spp. fluoroquinolone resistance was detected at different levels in the participating hospitals (at least 26% from *E. coli* and 20% from *Klebsiella spp.*). In the study on community-acquired urinary tract infections, Moghnieh et al. reported a similarly elevated level of fluoroquinolone resistance in *E. coli* and *Klebsiella* spp. (42 and 33%, respectively) [24]. This high percentage of fluoroquinolone resistance can be attributed to the misuse of this class of antibiotics since it is available over-the-counter in Lebanese community pharmacies [25, 26].

Regarding susceptibility of *Enterobacteriaceae* to carbapenems, the upper limit of the range was 100% in *E. coli* and *Klebsiella* spp.. So, there were hospitals that still reported 100% susceptibility, 2 of them for both *E. coli* and *Klebsiella* spp. and 1 for *Klebsiella* spp*.* and not *E. coli*. The rest of the hospitals, 11 in case of *E. coli* and 10 in *Klebsiella* spp., reported different levels of carbapenem resistance ranging from 1 to 5% in *E. coli* and 1 to 10% in *Klebsiella* spp. Two hospitals additionally reported carbapenemase production among their isolates. Hammoudi et al., in their multicentre cross-sectional study mentioned earlier, showed that the mean prevalence of carbapenem resistance in *Eneterobacteriaceae* was 1.2% [6]. The spread of carbapenem-resistant Enterobacteriaceae (CRE) in Lebanese hospitals is still lower than resistance to 3GC or fluroquinolones and carbapenem resistance in Enterobacteriaceae is predominantly due to OXA-48-like carbapenemases production [6, 23, 24]. As for risk factors for CRE infection, Kanafani et al. found that the respiratory tract and blood as primary infection sites and prior antibiotic use in the hospital setting were independent risk factors for its nosocomial acquisition [27]. There is a plausible risk for the transmission of resistant pathogens from the hospital environment into the community in the absence of rigorous infection control practices, efficient sanitation and sewage disposal and in a setting where antimicrobials are used in veterinary medicine without control. Daoud et al. confirmed this possibility when they detected a New Delhi metallo-beta-lactamase-1-producing *Enterobacteriaceae* strain in sewage water [28].

### Changing trends in antibiotic susceptibility compared with the 2011–2013 report

The “WHO AMR study group” listed 13 organism/antibiotic combinations, other than drug-resistant *Mycobacterium tuberculosis*, to be of high priority for discovery, research and development of new antibiotics [1]. In our study, 6 of these priority organisms have been described: 3GC-resistant *Enterobacteriaceae*, carbapenem-resistant *Enterobacteriaceae*, carbapenem-resistant *P. aeruginosa*, carbapenem-resistant *Acinetobacter* spp., fluoroquinolone-resistant *Salmonella* spp., and methicillin resistant *S. aureus*. Other organisms stated in the “WHO AMR study group” priority list were not included for the following reasons: lack of data as in the case of *M. tuberculosis*, a very small number of isolates of certain species reported by Lebanese laboratories such *Campylobacter* spp. and *Shigella* spp., and non-standardised methodologies used in the Lebanese laboratories like in reporting penicillin susceptibility in *S. pneumoniae* and vancomycin susceptibility pooling in *Enterococci* without differentiating between *E. faecalis* and *E. faecium*. When the 2015–2016 antibiotic susceptibility results of these 6 priority organisms were compared to those of 2011–2013, a statistically significant decrease in susceptibility was demonstrated (Fig. 3).

The emergence of antibiotic resistance has been strongly correlated to the inappropriate prescribing of antibiotics [29]. In Lebanon, there are no national surveillance studies describing antibiotic consumption on hospital setting, rather than very few hospital data showing that carbapenems are among the most commonly prescribed antibiotics [30, 31]. On the other hand, the extensive use of over-the-counter antibiotics is of paramount importance in aggravating the antimicrobial resistance. In Lebanon, the prevalence of dispensing broad-spectrum antibiotics without prescription ranged from 32 to 42% among community pharmacies [25, 26, 32]. This situation calls for immediate action against these practices through improving public and healthcare professional awareness on AMR, establishing and implementing rigorous legislation that forbids the dispensing of antibiotics over the counter, and establishing antimicrobial stewardship programmes in hospitals and the community. Antibiotics are not just used in humans, yet they are heavily administered for therapeutic and prophylaxis purposes in veterinary medicine, livestock production, crop culture and food production industry [33]. In Lebanon, there are no national data describing antibiotic use in the listed fields outside human health yet AMR has already emerged and is spreading [34]. This necessitates adopting multifaceted, comprehensive, and integrated measures complying with the “One Health” approach to curb the emergence and spread of AMR, and preserve the efficacy of the remaining few active antibiotics [33].

#### Comparative epidemiology with European data on AMR

Lebanon is a relatively small Arab country in the Eastern Mediterranean region. In international reports that present AMR rates on the global map, Lebanon, among many other countries of the Eastern Mediterranean region, is not included due to the lack of national surveillance data. A recently published narrative review of literature showed the significantly increasing prevalence of antibacterial resistance in different countries of the Arab World including Lebanon [3]. In the absence of official standardized AMR surveillance networks in neighbouring Arab countries, we chose to compare our data with those from EARS-Net aiming to position Lebanon on the global map of AMR, and taking into account the geographical proximity between Lebanon and several Southern and South-eastern European countries. The EARS-Net annual AMR surveillance reports provide an accurate picture of the extent of AMR in the European Union (EU) [11, 12]. We believe that comparing AMR in Lebanon to that from relatively nearby countries in Europe would help the reader to visualize and benchmark the AMR situation in Lebanon. This comparison would, most importantly, help Lebanese professionals and officials visualize the actual size of the problems caused by AMR and would hence stimulate them for serious interventions. Moreover, it could trigger international organisations like the WHO, the Food and Agriculture Organization of the United Nations, the World Organization for Animal Health, and others, to include Lebanon in global actions plans under the “One Health” approach that aim at limiting the spread and emergence of AMR.

Upon reviewing European and Lebanese data using percent susceptibility instead of resistance (Additional files 8, 9, 10, 11, 12, 13, 14 and 15), our data was comparable to those from countries in Southern and South-eastern Europe including Bulgaria, Romania, Greece, Italy and Spain that reported in general low antibiotic susceptibility patterns, in comparison with the countries of North and West Europe [11, 12]. In Lebanon, antimicrobials are dispensed over-the-counter in pharmacies and antimicrobial stewardship practices exist only in very few university hospitals [25, 26, 31, 32]. As for infection prevention and control (IPC) programmes, their presence is mandatory in different healthcare facilities. However, the monitoring of its function is variable across these facilities. At the national level, the assessment of the appropriateness of IPC practices is limited to the accreditation audit of done by the Lebanese Ministry of Health every 3 years. Recently a National Action Plan in line with the WHO Global Action Plan for fighting AMR is being prepared in Lebanon in collaboration with the ministries of health, agriculture and environment. This plan is directly supported by the WHO and its outcome may be reflected in future AMR surveillance studies. Joint efforts from representatives of the government and healthcare authorities are needed to ensure the effectiveness of this plan for the upcoming years.

Regarding IPC practices in hospitals in North and West Europe, surveillance of hospital-acquired infections and IPC practices is present through the establishment of national key performance indicators, which reflects a serious collaborative work in infection control [35–38]. In addition, antibiotic consumption in hospitals and the community, in these countries, is much lower than others from South and East Europe, as reported to the European Surveillance of Antimicrobial Consumption Network (ESAC-Net) [39]. In 2015, Greece and Romania were the countries with the highest consumption of antibacterials for systemic use in Europe [39]. In addition, despite that components of IPC infrastructure are in place in hospital setting in several Southern and South-eastern countries, gaps leading to suboptimal IPC are present such as: insufficient time allocated to IPC activities by healthcare givers and personnel due to understaffing, considerable variation in safety culture for effective IPC among different facilities, inadequate collaboration between healthcare professionals at different levels, and absence of documented process audits that assess compliance with IPC practices [40–44]. However, European countries like Spain, Italy, Bulgaria and Romania, among others, are in the process of implementing their own national action plans for the control of AMR, in close collaboration with the European Centre for Disease Prevention and Control (ECDC) [40–44].

#### Limitations and strengths

Despite the national effort to improve reporting of antibiotic susceptibility in Lebanon, there is still a need for implementing uniform standardised microbiological methods and adequate quality control systems in all Lebanese laboratories. The lack of both elements in majority of Lebanese hospitals has led to limiting the number of included centres to only 13 of the 152 governmental and private hospitals in Lebanon. Another limitation is the lack of organism stratification as per type of clinical specimen or per its site of acquisition, i.e. nosocomial or community. However, this report may serve as an example for countries that do not have standardised national surveillance for AMR. This study and the one published earlier allow Lebanon to be included in the global AMR maps. Furthermore, generating local antibiograms is critical for establishing national clinical guidelines for the management of infectious diseases.

## Conclusion

The current compiled antibiotic susceptibility data have shed light on increasing bacterial resistance trends in Lebanon, which were found to be comparable with data from some Eastern and Southern European countries. However, there is a need for a robust national AMR surveillance system in Lebanon, which could be included in the already present global systems for providing accurate and reliable data. Moreover, there is a need for a multifaceted programme for AMR containment based on the “One Health” approach. This programme should include the implementation of rigorous infection prevention and control programmes in hospitals, antimicrobial stewardship programmes in both hospitals and the community and the control of antibiotic use in livestock and agriculture. Furthermore, raising awareness about the extent, implications and modalities of preventing AMR in Lebanon should be performed for healthcare providers and the public. Finally, research for developing alternative therapies for infectious diseases, such as phage therapy and microbiota-based therapy, should be encouraged to limit the abuse and subsequently the loss of effectiveness of available antimicrobials.

### Key findings

- Microbiological methods used for AMR detection in Lebanese hospitals majorly conformed to the CLSI and EUCAST guidelines on antibiotic susceptibility testing.
- Non-uniformity in AMR reporting was observed in some cases, such as susceptibilities of *Acinetobacter* spp. to colistin, *S. pneumoniae* to penicillin and *Salmonella* spp. to fluoroquinolone.
- A wide range of susceptibility results was reported for each antibiotic–microbe combination among the 13 laboratories.
- Fluoroquinolone resistance in *Enterobacteriaceae*, including *Salmonella* spp., has been established in the Lebanese community.
- Lebanese hospitals have become endemic for 3GC resistant-*Enterobacteriaceae*, and this resistance pattern is apparently finding its way to the community.
- Some hospitals laboratories reported the emergence of carbapenem-resistant *Enterobacteriaceae* and *P. aeruginosa*, along with established and increasing carbapenem resistance in *Acinetobacter* spp.
- Among Gram positive bacteria, *S. aureus* mean percent susceptibility to methicillin was 72%, and that of *Enterococci* to ampicillin was 75%.
- Susceptibility of *S. pneumoniae* to penicillin reached a mean of 75% from all tested isolates in 3 of the 13 hospitals in addition to the emergence of levofloxacin-resistant strains in some centres.
- Despite the relatively short time between the 2011–2013 and 2015–2016 compilation, a trend towards decreasing antibiotic susceptibility of less than 10% was observed among several clinically important bacteria.
- The 2015–2016 Lebanese data were found to be comparable with those from Eastern and South-eastern European countries, such as Greece, Spain, Italy and Bulgaria. These countries are in the process of developing their own national action plans to fight AMR supported by the ECDC.
- In Lebanon, a national action plan for combating AMR s being prepared in collaboration with representatives of government, healthcare authorities and is supported directly by the WHO.

## Additional files

Additional file 1: **Table S1.** The microbiological methods used in detecting certain resistance patterns in the included Lebanese hospitals and the corresponding Clinical and Laboratory Standards Institute (CLSI)/ European Committee on Antimicrobial Susceptibility Testing (EUCAST) guidelines recommendations. (DOCX 131 kb)

Additional file 2: Formulas for mean percent (%) susceptibility calculation. (DOCX 121 kb)

Additional file 3: **Table S1.** *E. coli* and *Klebsiella spp* percent susceptibility* to antibiotics in 13 Lebanese hospitals during 2015/2016. (DOCX 121 kb)

Additional file 4: **Table S1.** *Pseudomonas aeruginosa* and *Acinetobacter* spp. ^a^ percent susceptibility* to antibiotics in 13 Lebanese hospitals during 2015/2016. (DOCX 129 kb)

Additional file 5: **Table S1.** *Salmonella* spp. percent susceptibility* to antibiotics in 10 Lebanese hospitals during 2015/2016. (DOCX 70 kb)

Additional file 6: **Table S1.** *Staphylococcus aureus*, coagulase negative *Staphylococci* ^a^, and *Enterococcus spp.* percent susceptibility* to antibiotics in 13 Lebanese hospitals during 2015/2016. (DOCX 115 kb)

Additional file 7: **Table S1.** *Streptococcus pneumoniae, Streptococcus viridans, Streptococcus pyogenes*, and *Streptococcus agalactiae* percent susceptibility* to antibiotics in 13 Lebanese hospitals during 2015/2016 ^a^. (DOCX 103 kb)

Additional file 8: **Table S1.** *E. coli* percent susceptibility to third-generation cephalosporins in countries of the European Union, based on the 2015 and 2016 annual reports of the European Antimicrobial Resistance Surveillance Network (EARS-Net)^1,2^, and comparison to 2015–2016 Lebanese data. (DOCX 110 kb)

Additional file 9: **Table S1.** *E. coli* percent susceptibility to carbapenems in countries of the European Union, based on the 2015 and 2016 annual reports of the European Antimicrobial Resistance Surveillance Network (EARS-Net)^1,2^, and comparison to 2015–2016 Lebanese data. (DOCX 113 kb)

Additional file 10: **Table S1.** *K. pneumoniae* percent susceptibility to third-generation in countries of the European Union, based on the 2015 and 2016 annual reports of the European Antimicrobial Resistance Surveillance Network (EARS-Net)^1,2^, and comparison to 2015–2016 Lebanese data. (DOCX 110 kb)

Additional file 11: **Table S1.** *K. pneumoniae* percent susceptibility to carbapenems in countries of the European Union, based on the 2015 and 2016 annual reports of the European Antimicrobial Resistance Surveillance Network (EARS-Net)^1,2^, and comparison to 2015–2016 Lebanese data. (DOCX 106 kb)

Additional file 12: **Table S1.** *P. aeruginosa* percent susceptibility to carbapenems in countries of the European Union, based on the 2015 and 2016 annual reports of the European Antimicrobial Resistance Surveillance Network (EARS-Net)^1,2^, and comparison to 2015–2016 Lebanese data. (DOCX 114 kb)

Additional file 13: **Table S1.** *Acinetobacter* spp. percent susceptibility to carbapenems in countries of the European Union, based on the 2015 and 2016 annual reports of the European Antimicrobial Resistance Surveillance Network (EARS-Net)^1,2^, and comparison to 2015–2016 Lebanese data. (DOCX 112 kb)

Additional file 14: **Table S1.** *S. aureus* percent susceptibility to methicillin in countries of the European Union during 2015 and 2016, based on the 2015 and 2016 annual reports of the European Antimicrobial Resistance Surveillance Network (EARS-Net)^1,2^, and comparison to 2015–2016 Lebanese data. (DOCX 111 kb)

Additional file 15: **Table S1.** *S. pneumonaie* percent susceptibility to penicillin countries of the European Union during 2015 and 2016, based on the 2015 and 2016 annual reports of the European Antimicrobial Resistance Surveillance Network (EARS-Net)^1,2^, and comparison to 2015–2016 Lebanese data. (DOCX 110 kb)

## Abbreviations

3GC: Third-generation cephalosporins
AMR: Antimicrobial resistance
AST: Antimicrobial susceptibility testing
BMD: Broth microdilution
CLSI: Clinical & Laboratory Standards Institute
CRE: Carbapenem-resistant *Enterobacteriaceae*
DD: Disc diffusion
EARS-Net: European Antimicrobial Resistance Surveillance Network
ECDC: European Centre for Disease Prevention and Control
ESAC-Net: European Surveillance of Antimicrobial Consumption Network
ESBL: Extended-spectrum beta-lactamase
EU: European Union
EUCAST: European Committee on Antimicrobial Susceptibility Testing
GLASS: Global Antimicrobial Resistance Surveillance System
IPC: Infection prevention and control
IRB: Institutional review board
MIC: Minimal inhibitory concentration
OR: Odd ratio
WHO: World Health Organization
