# Supplementary material for: A compilation of antimicrobial susceptibility data from a network of 13 Lebanese hospitals reflecting the national situation during 2015–2016
Source: Antimicrob Resist Infect Control. 2019 Feb 20;8:41. doi: 10.1186/s13756-019-0487-5 (PMC6381724; doi:10.1186/s13756-019-0487-5)
Supplement: Supplementary file 1 — Table S1. The microbiological methods used in detecting certain resistance patterns in the included Lebanese hospitals and the corresponding Clinical and Laboratory Standards Institute (CLSI)/ European Committee on Antimicrobial Susceptibility Testing (EUCAST) guidelines recommendations. (DOCX 131 kb) [file 13756_2019_487_MOESM1_ESM.docx]

**Additional file 1**

**Table 1.** The microbiological methods used in detecting certain resistance patterns in the included Lebanese hospitals and the corresponding Clinical and Laboratory Standards Institute (CLSI)/ European Committee on Antimicrobial Susceptibility Testing (EUCAST) guidelines recommendations

| **Microbiological Methods** | **CLSI guidelines Recommendations [1,2]** | **EUCAST guidelines Recommendations [3-5]** | **Methods reported by Lebanese hospitals (n=13)** |
| --- | --- | --- | --- |
| **Detection/Screening of 3GC susceptibility in *Enterobacteriaceae*** | -CRO or CTX MIC or DD methods  CRO or CTX MIC breakpoints  -S: ≤ 1 mcg/mL, I: 2 mcg/mL, R: ≥ 4 mcg/mL  CRO DD breakpoints  -S: *d* ≥ 23 mm, I: *d* = 20-22 mm, R: *d* ≤ 19 mm)  CTX DD breakpoints  -S: *d* ≥ 26 mm, I: *d* = 23-25 mm, R: *d* ≤ 22 mm)  -When using the above criteria, routine ESBL testing is no longer necessary. | -CTX MIC or DD methods  MIC breakpoints  S: ≤ 1 mcg/mL, R: > 2 mcg/mL  DD breakpoints  -S: *d* ≥ 20 mm, R: *d* < 19 mm)  -These breakpoints for will detect all clinically important resistance mechanisms.  -Some isolates that produce BLs are 'S' or 'I' to 3GC or 4GC with these breakpoints and should be reported as tested. | Number of reporting hospitals = 13  -Automated MIC method: 5 (2/5 hospitals test only one 3GC and extrapolate the susceptibility to the others, 3/5 hospitals report the susceptibility for each 3GC alone)  -DD method: 6 hospitals (2/6 hospitals test only one 3GC and extrapolate the susceptibility to the others)  -2 hospitals determined ESBL production, and extrapolated 3GC susceptibility from it. |
| **Detection of ESBL production in *Enterobacteriaceae*** | -If screening is positive, the confirmatory test must be performed to establish the presence of an ESBL.  -Combination disk test or BMD (cephalosporin ± clavulanic acid)  -DD method (+) result:  ≥ 5 mm increase in *d* of the combination (cephalosporin + clavulanic acid) compared to the cephalosporin alone  -MIC method (+) result:  ≥ 3 twofold decrease in MIC of the combination (cephalosporin + clavulanic acid) compared to the cephalosporin alone  -ESBL testing may be useful for epidemiological or infection control purposes. | -Combination disk test (mostly recommended), ESBL gradient test, BMD (cephalosporin ± clavulanic acid)  -Double-disk synergy test (3GC or 4GC disk + AMC disk)  -DD method (+) result:  ≥ 5 mm increase in inhibition zone.  -MIC method or ESBL gradient test (+) result:  MIC ratio ≥ 8  - Double-disk synergy test (+) result: expansion of indicator cephalosporin inhibition zone towards AMC disk  -ESBL detection and characterization recommended for public health and infection control purposes | Number of reporting hospitals = 8 (in concordance with CLSI recommendations) |
| **Detection/Screening of carbapenem susceptibility in *Enterobacteriaceae*** | Carbapenem MIC or DD methods  -ETP MIC breakpoints:  S: ≤0.5 mcg/mL, I: 1 mcg/mL, R: ≥2 mcg/mL  -ETP DD breakpoints:  S: *d* ≥ 22 mm, R: *d* ≤ 18 mm  -IPM/ MEM MIC breakpoints:  S: ≤ 1 mcg/mL, I: 2 mcg/mL, R: ≥ 4 mcg/mL  -IPM/ MEM DD breakpoints:  S: *d* ≥ 23 mm, R: *d* ≤ 19 mm  -When using the above criteria, testing carbapenemase production is no longer necessary. | Carbapenem MIC or DD methods  -ETP MIC breakpoints:  S: ≤ 0.5 mcg/mL, R: > 1 mcg/mL  -ETP DD breakpoints:  S: *d* ≥25 mm, R: *d* < 22 mm  -IPM/ MEM MIC breakpoints:  S: ≤ 2 mcg/mL, R: ≥ 8 mcg/mL  -IPM/ MEM DD breakpoints:  S: *d* ≥ 22 mm, R: *d* < 16 mm  -These breakpoints for will detect all clinically important resistance mechanisms.  -Some isolates that produce carbapenemase are categorised as 'S' with these breakpoints and should be reported as tested. | Number of reporting hospitals = 13  -7 hospitals tested the susceptibility to each carbapenem using either automation or DD method  -4 hospitals performed ETP E-test screening and extrapolated from IPM and MEM susceptibility.  -2 hospitals proceeded to further phenotypic testing using MHT* or EDTA/PDA ± CXA |
| **Detection of carbapenemase production in *Enterobacteriaceae*** | -If screening is positive, the confirmatory test must be performed to establish the presence of a carbapenemase.  -MHT*, Carba NP test, and/or a molecular assay when isolates of Enterobacteriaceae are suspicious for carbapenemase production based on IPM/MEM MICs of 2-4 mcg/mL or ETP MIC of 2 mcg/mL  -MHT recommended for epidemiological or infection control purposes only | -Combination disk testing essays (MEM ± inhibitors such as APBA and PBA)  -Carba NP test  -Carbapenemase detection and characterization recommended for public health and infection control purposes only | Number of reporting hospitals = 3 (using MHT) |
| **Detection of fluoroquinolone susceptibility in *Salmonella spp.*** | -CIP MIC method  -S: ≤ 0.06 mcg/mL, I: 0.12-0.5 mcg/mL, R: ≥ 1 mcg/mL  -PEFLOX DD method  -S: *d* ≥ 24 mm, R: *d* ≤ 23 mm)  -When PEFLOX disc is NA, use either CIP DD method (S: *d* ≥ 31 mm, R: *d* ≤ 20 mm) or NAL DD method (S: *d* ≥19 mm, R: *d* ≤16 mm). | -CIP MIC method:  -S: ≤ 0.06 mcg/mL, R: ≥ 0.06 mcg/mL  -PEFLOX DD method:  -S if *d* ≥ 24 mm, R if *d* ≤ 23 mm) | Number of reporting hospitals = 10  -NAL automated MIC or DD methods: 5 hospitals  -CIP automated MIC or DD methods: 3 hospitals  -PEFLOX DD method: 2 hospitals |
| **Detection of penicillin susceptibility in *S. pneumoniae*** | -OXA DD method  -S: *d* ≥ 20 mm  -*d* < 20 mm 🡪 perform PEN MIC  -PEN MIC breakpoints  *-Meningitis breakpoints*  S: ≤ 0.06 mcg/mL, R: ≥ 0.12 mcg/mL  *-Non meningitis breakpoints*  S: ≤2 mcg/mL, I: 4 mcg/mL, R: ≥8 mcg/mL | -OXA DD method  -*d* < 20 mm 🡪 perform benzylpenicillin MIC  -Benzylpenicillin MIC breakpoints  *-Infections other than meningitis breakpoints:*  S: ≤0.06 mcg/mL, R: >2 mcg/mL  -*Benzylpenicillin meningitis:*  S: ≤0.06 mcg/mL, R: >0.06 mcg/mL | Number of reporting hospitals = 8  -OXA DD method (starting point): 8 hospitals  -OXA DD method only (no MIC method): 4 hospitals  -PEN automated MIC method and interpretation using meningitis breakpoints: 3 hospitals  -One hospital performed PEN E-test then reported susceptibility at MIC < 1 mcg/ml, and resistance at MIC > 2 mcg/mL |
| **Detection of MRSA** | -OXA MIC method or FOX DD method  -OXA breakpoints  -S: ≤ 2 mcg/mL, R: ≥ 4 mcg/mL  -FOX breakpoints  -S: *d* ≥ 22 mm, R: *d* ≤ 21 mm | -OXA MIC method or FOX DD method  -OXA breakpoints: R: > 2 mcg/mL  -FOX breakpoints:  -S: *d* ≥ 22 mm, R: *d* < 22 mm | -Number of reporting hospitals = 13  -FOX DD method: 7 hospitals  -FOX automated MIC method: 2 hospitals  -OXA automated MIC method: 3 hospitals  -OXA DD method: 1 hospital |
| **Detection of colistin susceptibility in *P. aeruginosa*** | -CST MIC or DD methods  -MIC breakpoints  -S: ≤ 2 mcg/mL, I: 4 mcg/mL, R: ≥ 8 mcg/mL  -DD breakpoints  S: *d* ≥11 mm, R: *d* ≤10 mm | -CST MIC method  -MIC breakpoints  S: ≤ 2 mcg/mL, R: > 2 mcg/mL | -Number of reporting hospitals = 13  -CST automated MIC method: 5 hospitals  -CST DD method: 8 hospitals |
| **Detection of colistin susceptibility in *Acinetobacter spp.***** | -CST MIC method (DD method is not recommended)  **-**MIC breakpoints  S: ≤ 2 mcg/mL, R: ≥ 4 mcg/mL | -CST MIC method  **-**MIC breakpoints  S: ≤ 2 mcg/mL, R: > 2 mcg/mL | -Number of reporting hospitals = 11  -CST automated MIC method: 6 hospitals  -CST DD method: 5 (3 hospitals use a *d* of 11 mm as a breakpoint, while 2 hospitals use 15 mm as a breakpoint) |

**KEY: AMC** = amoxicillin/clavulanic acid, **APBA**=aminophenyl boronic acid, **BL** = Beta-lactamases, **BMD** = brothmicrodilution, **CIP** = Ciprofloxacin, **CLSI** = Clinical and Laboratory Standards Institute, **CRO** = Ceftriaxone, **CST** = Colistin, **CTX** = Cefotaxime, **CXA** = Cloxacillin, ***d*** = Diameter, **DD** = Disc Diffusion, **EUCAST** = European Committee on Antimicrobial Susceptibility Testing, **EDTA** = Ethylenediaminetetraacetic acid, **ESBL**= Extended Spectrum Beta-Lactamase, **ETP** = Ertapenem, **FOX** = cefoxitin, **I** = Intermediate, **IPM** = Imipenem, **MEM** = Meropenem, **MHT** = modified Hodge test, **MIC** = Minimum Inhibitory Concentration, **MRSA** = methicillin resistant *S. aureus*, **NA** = Not Available, **NAL** = Nalidixic Acid, **OXA** = Oxacillin, **PBA**=phenyl boronic acid, **PEFLOX** = Pefloxacin, **PEN** = Penicillin, **R** = Resistant, **S** = Susceptible, **3GC**= third-generation cephalosporin, **4GC**= forth-generation cephalosporin, **(+)** = positive.

**N.B.** * Refer to the 2015 CLSI guidelines for further information on MHT.

** In this study, we excluded the results related to *Acinetobacter spp.* susceptibility to colistin since none of the included centres abided by the CLSI and EUCAST guidelines.

**References**

1. CLSI. *Performance Standards for Antimicrobial Susceptibility Testing; Twenty-Fifth Informational Supplement.* CLSI document M100-S25. Wayne, PA: Clinical and Laboratory Standards Institute; 2015.
2. CLSI. *Performance Standards for Antimicrobial Susceptibility Testing; Twenty-Sixth Informational Supplement.* CLSI document M100-S26. Wayne, PA: Clinical and Laboratory Standards Institute; 2016.
3. The European Committee on Antimicrobial Susceptibility Testing. Breakpoint tables for interpretation of MICs and zone diameters. Version 5.0, 2015. http://www.eucast.org.
4. The European Committee on Antimicrobial Susceptibility Testing. Breakpoint tables for interpretation of MICs and zone diameters. Version 6.0, 2016. http://www.eucast.org.
5. Giske CG, Martinez L, Cantón R, Stefani S, Skov R, Glupczynski Y, et al. 2013. EUCAST guidelines for detection of resistance mechanisms and specific resistances of clinical and/or epidemiological importance. <http://www.eucast.org/fileadmin/src/media/PDFs/EUCAST_files/Resistance_mechanisms/EUCAST_detection_of_resistance_mechanisms_v1.0_20131211.pdf>.
