# Supplementary material for: A compilation of antimicrobial susceptibility data from a network of 13 Lebanese hospitals reflecting the national situation during 2015–2016
Source: Antimicrob Resist Infect Control. 2019 Feb 20;8:41. doi: 10.1186/s13756-019-0487-5 (PMC6381724; doi:10.1186/s13756-019-0487-5)
Supplement: Supplementary file 2 — Formulas for mean percent (%) susceptibility calculation. (DOCX 121 kb) [file 13756_2019_487_MOESM2_ESM.docx]

**Additional file 2**

Formulas for mean percent (%) susceptibility calculation

Formula for mean percent (%) susceptibility of specific bacteria (A) to a specific antibiotic (B) from the included Lebanese laboratories (N=13)

*For Laboratory number 1:*

- Number of “A” isolates susceptible to “B” in 2015 from “Laboratory number 1” (C1) = (% susceptibility of “A” to “B” in 2015 × number of tested “A” isolates in 2015) ⁄ 100
- Number of “A” isolates susceptible to “B” in 2016 from “Laboratory number 1” (D1) = (% susceptibility of “A” to “B” in 2016 × number of tested “A” isolates in 2016) ⁄ 100
- Total number of “A” isolates susceptible to “B” in 2015/2016 from “Laboratory number 1” (E1) = C1 + D1
- Total number of tested “A” isolates during 2015 and 2016 from “Laboratory number 1” (F1) = number of tested “A” isolates in 2015 + number of tested “A” isolates in 2016

For the rest of laboratories till Laboratory number 13, we calculate the same parameters in the same way.

*For the 13 Laboratories*

Mean % susceptibility of “A” to “B” during 2015/2016 = ($\sum_{i=1}^{13} Ei$ $\div$ $\sum_{i=1}^{13} Fi$) $\times$ 100

Formula for mean percent (%) susceptibility of specific bacteria (A) to a specific antibiotic (B) in a specific European country (C) during 2015/2016

In the 2015 and 2016 European antimicrobial resistance surveillance reports [1,2], the data was reported as % resistance. So, we used this data to calculate % susceptibility.

-% Susceptibility of “A” to “B” in “C” in 2015 (D) = 100 − % Resistance of “A” to “B” in “C” in 2015

-% Susceptibility of “A” to “B” in “C” in 2016 (E) = 100 − % Resistance of “A” to “B” in “C” in 2016

-Number of “A” isolates susceptible to “B” in “C” in 2015 (F) = (D × number of tested “A” isolates in “C” in 2015) ⁄ 100

-Number of “A” isolates susceptible to “B” in “C” in 2016 (G) = (E × number of tested “A” isolates in “C” in 2016) ⁄ 100

-Total number of tested (A) isolates in (C) during 2015 and 2016 (H) = number of tested “A” isolates in “C” in 2015 + number of tested “A” isolates in “C” in 2016

-Mean % susceptibility of “A” to “B” in “C” during 2015/2016 = (F+ G) ⁄ H ×100.

Example:

Mean % susceptibility of *K. pneumoniae* to carbapenems in France during 2015/2016

-% Susceptibility of *K. pneumoniae* to carbapenems in France in 2015 = 100 – 0.5 = 95.5

-% Susceptibility of *K. pneumoniae* to carbapenems in France in 2016 = 100 – 0.4 = 95.6

-Number of *K. pneumoniae* isolates susceptible to carbapenems in France in 2015 = (95.5× 2244) ⁄ 100 = 2143

-Number of *K. pneumoniae* isolates susceptible to carbapenems in France in 2016 = (95.6 × 2528) ⁄ 100 = 2417

-Total number of tested *K. pneumoniae* isolates in France during 2015 and 2016 = 2244 + 2528 = 4772

-Mean % susceptibility of *K. pneumoniae* to carbapenems in France during 2015/2016 = (2143+ 2417) ⁄ 4772 ×100 = 95.6

References

1. European Centre for Disease Prevention and Control. Antimicrobial resistance surveillance in Europe 2015. Annual Report of the European Antimicrobial Resistance Surveillance Network (EARS-Net). Stockholm: ECDC; 2016.
2. European Centre for Disease Prevention and Control. Antimicrobial resistance surveillance in Europe 2016. Annual Report of the European Antimicrobial Resistance Surveillance Network (EARS-Net). Stockholm: ECDC; 2017.
