# Supplementary material for: A compilation of antimicrobial susceptibility data from a network of 13 Lebanese hospitals reflecting the national situation during 2015–2016
Source: Antimicrob Resist Infect Control. 2019 Feb 20;8:41. doi: 10.1186/s13756-019-0487-5 (PMC6381724; doi:10.1186/s13756-019-0487-5)
Supplement: Supplementary file 3 — Table S1. E. coli and Klebsiella spp percent susceptibility* to antibiotics in 13 Lebanese hospitals during 2015/2016. (DOCX 121 kb) [file 13756_2019_487_MOESM3_ESM.docx]

**Additional file 3**

## Table 1. *E. coli* and *Klebsiella spp* percent susceptibility* to antibiotics in 13 Lebanese hospitals during 2015/2016

| **Antibiotics** | ***E. coli*** | | ***Klebsiella* spp** | |
| --- | --- | --- | --- | --- |
|  | **No. of tested isolates** | **% S**  **(Range)** | **No. of tested isolates** | **% S (Range)** |
| Amikacin | 41818 | 90 (70-100) | 9498 | 94 (66-99) |
| Amoxicillin/clavulanic acid | 39116 | 59 (42-72) | 9069 | 62 (42-80) |
| Ampicillin | 35906 | 24 (12-31) | NR | NR |
| Aztreonam | 40029 | 57 (22-73) | 7924 | 65 (37-76) |
| Cefepime | 40524 | 63 (47-74) | 9292 | 65 (38-76) |
| Cefoxitin | 33104 | 76 (58-95) | 6373 | 83 (53-93) |
| Ceftazidime | 41816 | 62 (51-72) | 9498 | 64 (39-76) |
| Ceftriaxone | 41816 | 58 (47-70) | 9498 | 63 (35-72) |
| Cefuroxime | 33106 | 56 (30-66) | 6579 | 61 (27-68) |
| Ciprofloxacin | 40524 | 57 (44-74) | 9311 | 71 (56-80) |
| Ertapenem | 19932 | 97 (96-100) | 5290 | 94 (86-100) |
| Fosfomycin | 21086 | 97 (57-99) | NR | NR |
| Gentamicin | 41818 | 72 (41-92) | 9498 | 78 (61-85) |
| Imipenem | 41813 | 97 (95-100) | 9498 | 96 (90-100) |
| Meropenem | 8775 | 99 (96-100) | 1410 | 97 (90-100) |
| Nitrofurantoin | 29434 | 87 (60-98) | 5155 | 59 (30-71) |
| Piperacillin/tazobactam | 40524 | 76 (47-90) | 9292 | 78 (46-90) |
| Tigecycline | 39050 | 96 (67-100) | 8546 | 93 (61-100) |
| Trimethoprim/sulfamethoxazole | 41496 | 53 (40-65) | 9498 | 58 (45-74) |
| ESBL-production (prevalence) ^a^ | 19410 | 34.5 (29.5-39) | 5215 | 33 (25-43) |
| CRE (prevalence) ^a^ | 2539 | 0.6 (0.3-3.1) | 453 | 2 (1-9.6) |

**Key=** CRE: Carbapenem-resistant Enterobacteriaceae, ESBL: Extended-spectrum beta-lactamase, NR: not reported, S: Susceptibility, %: Percentage.

**N.B.**

^a^ The prevalence of ESBL-producing species was extracted from 8 hospitals and that of CRE from 2 hospitals. The values did not depend on antibiogram results, yet on other detection methods recommended by the CLSI and EUCAST guidelines.

*Susceptibility is represented as mean (%) for each antibiotic-microbe combination and the range is the upper and lower limits of individual % susceptibility from participating centres.
