# Supplementary material for: A compilation of antimicrobial susceptibility data from a network of 13 Lebanese hospitals reflecting the national situation during 2015–2016
Source: Antimicrob Resist Infect Control. 2019 Feb 20;8:41. doi: 10.1186/s13756-019-0487-5 (PMC6381724; doi:10.1186/s13756-019-0487-5)
Supplement: Supplementary file 4 — Table S1. Pseudomonas aeruginosa and Acinetobacter spp. a percent susceptibility* to antibiotics in 13 Lebanese hospitals during 2015/2016. (DOCX 129 kb) [file 13756_2019_487_MOESM4_ESM.docx]

**Additional file 4**

## Table 1. *Pseudomonas aeruginosa* and *Acinetobacter* spp ^a^ percent susceptibility* to antibiotics in 13 Lebanese hospitals during 2015/2016

| **Antibiotics** | ***P. aeruginosa*** | | ***Acinetobacter* spp** ^a^ | |
| --- | --- | --- | --- | --- |
|  | **No. of tested isolates** | **% S (Range)** | **No. of tested isolates** | **% S (Range)** |
| Amikacin | 9005 | 85 (77-96) | 3675 | 19 (9-74) |
| Aztreonam | 7457 | 79 (43-91) | NR^b^ | NR^b^ |
| Cefepime | 9005 | 81 (61-91) | 3675 | 13 (2-67) |
| Ceftazidime | 9005 | 80 (38-94) | 3675 | 13 (2-60) |
| Ciprofloxacin | 9005 | 73 (53-88) | 3675 | 11 (3-76) |
| Colistin^c^ | 5440 | 98 (81-100) | NA | NA |
| Gentamicin | 9005 | 81 (72-95) | 3675 | 14 (9-74) |
| Imipenem | 9005 | 70 (55-95) | 3675 | 12 (3-74) |
| Meropenem | 3256 | 69 (45-95) | 225 | 33 (17-74) |
| Piperacillin/tazobactam | 9005 | 78 (42-93) | 3675 | 11 (2-63) |
| Tigecycline^d^ | NA | NA | 3401 | 80 (29-97) |
| Trimethoprim/sulfamethoxazole | NR | NR | 3509 | 12 (6-42) |

**Key=** NA: not applicable, NR: not reported, S: Susceptibility, %: Percentage.

**N.B.** Susceptibility is represented as mean (%) for each antibiotic-microbe combination and the range is the upper and lower limits of individual % susceptibility from participating centers.

^a^ *Acinetobacter spp.* susceptibilities were obtained from 11 Lebanese hospitals.

^b^ *Acinetobacter* spp. susceptibility to aztreonam was not reported by the included laboratories during 2015/2016.

^c^ *Acinetobacter* spp. susceptibility to colistin was not calculated since none of the hospitals used the broth microdilution method as recommended by CLSI and EUCAST guidelines.

^d^ Tigecycline is not active against *Pseudomonas* spp.

*Susceptibility is represented as mean (%) for each antibiotic-microbe combination and the range is the upper and lower limits of individual % susceptibility from participating centres.
