# Supplementary material for: A compilation of antimicrobial susceptibility data from a network of 13 Lebanese hospitals reflecting the national situation during 2015–2016
Source: Antimicrob Resist Infect Control. 2019 Feb 20;8:41. doi: 10.1186/s13756-019-0487-5 (PMC6381724; doi:10.1186/s13756-019-0487-5)
Supplement: Supplementary file 5 — Table S1. Salmonella spp. percent susceptibility* to antibiotics in 10 Lebanese hospitals during 2015/2016. (DOCX 70 kb) [file 13756_2019_487_MOESM5_ESM.docx]

**Additional file 5**

**Table 1.** *Salmonella* spp percent susceptibility* to antibiotics in 10 Lebanese hospitals during 2015/2016

| **Antibiotics** | ***Salmonella* spp** | |
| --- | --- | --- |
|  | **No. of tested isolates** | **% S (Range)** |
| Ampicillin | 596 | 85 (73-92) |
| Ceftriaxone | 655 | 97 (96-100) |
| Cefotaxime | 561 | 98 (98-100) |
| Ceftazidime | 728 | 98 (96-100) |
| Trimethoprim/sulfamethoxazole | 721 | 71 (44-100) |
| Ciprofloxacin | 721 | 90 (56-100) |

**Key=** S: Susceptibility, %: Percentage.

*Susceptibility is represented as mean (%) for each antibiotic-microbe combination and the range is the upper and lower limits of individual % susceptibility from participating centres.
