# Supplementary material for: A compilation of antimicrobial susceptibility data from a network of 13 Lebanese hospitals reflecting the national situation during 2015–2016
Source: Antimicrob Resist Infect Control. 2019 Feb 20;8:41. doi: 10.1186/s13756-019-0487-5 (PMC6381724; doi:10.1186/s13756-019-0487-5)
Supplement: Supplementary file 6 — Table S1. Staphylococcus aureus, coagulase negative Staphylococci a, and Enterococcus spp. percent susceptibility* to antibiotics in 13 Lebanese hospitals during 2015/2016. (DOCX 115 kb) [file 13756_2019_487_MOESM6_ESM.docx]

**Additional file 6**

**Table 1.** *Staphylococcus aureus*, coagulase negative *Staphylococci* ^a^, and *Enterococcus spp.* percent susceptibility* to antibiotics in 13 Lebanese hospitals during 2015/2016

| **Antibiotics** | ***Staphylococcus aureus*^b^** | | ***Coagulase negative Staphylococci* ^a,b^** | | ***Enterococcus* spp ^b^** | |
| --- | --- | --- | --- | --- | --- | --- |
|  | **No. of tested isolates** | **% S (Range)** | **No. of tested isolates** | **% S (Range)** | **No. of tested isolates** | **% S (Range)** |
| Ampicillin^b^/ Oxacillin | 6452 | 72 (52-86) | 8472 | 39 (7-60) | 3760 | 75 (46-96) |
| Ciprofloxacin | 4083 | 81 (71-85) | 5115 | 44 (19-58) | NA^c^ | NA^c^ |
| Clindamycin | 6452 | 81 (31-97) | 8472 | 53 (37-83) | NR | NR |
| Erythromycin | 6304 | 73 (41-86) | 8472 | 32 (8-60) | 3730 | 30 (3-53) |
| Gentamicin | 3739 | 93 (88-97) | 4693 | 65 (42-80) | 1335 | 74 (58-93) |
| Levofloxacin | 2873 | 72 (57-99) | 3485 | 54 (20-97) | 2998 | 53 (14-85) |
| Linezolid | NR | NR | NR | NR | 694 | 99 (90-100) |
| Nitrofurantoin | 2117 | 87 (57-100) | 4396 | 81 (43-98) | NA^d^ | NA^d^ |
| Tetracycline | NR | NR | NR | NR | 1848 | 21 (13-41) |
| Tigecycline | 1788 | 99 (95-100) | 3702 | 83 (79-100) | 1022 | 99 (90-100) |
| Trimethoprim/sulfamethoxazole | 6437 | 88 (61-99) | 8472 | 60 (50-86) | NR | NR |
| Vancomycin | 6452 | 100 (96-100) | 8472 | 100 (79-100) | 3760 | 98 (83-100) |

**Key=** NA: not applicable, NR: not reported, S: Susceptibility, %: Percentage.

**N.B.**

^a^ Coagulase negative *Staphylococci* susceptibility were obtained from 12 Lebanese hospitals.

^b^ Susceptibility to oxacillin was tested in *Staphylococcus aureus* and coagulate negative *Staphylococci*, while susceptibility to ampicillin was tested in *Enterococcus spp*.

^c^ Ciprofloxacin is not active against *Enterococcus* spp.

^d^ Nitrofuratoin susceptibility is not tested against *Enterococcus* spp.

*Susceptibility is represented as mean (%) for each antibiotic-microbe combination and the range is the upper and lower limits of individual % susceptibility from participating centres.
