# Supplementary material for: A compilation of antimicrobial susceptibility data from a network of 13 Lebanese hospitals reflecting the national situation during 2015–2016
Source: Antimicrob Resist Infect Control. 2019 Feb 20;8:41. doi: 10.1186/s13756-019-0487-5 (PMC6381724; doi:10.1186/s13756-019-0487-5)
Supplement: Supplementary file 7 — Table S1. Streptococcus pneumoniae, Streptococcus viridans, Streptococcus pyogenes, and Streptococcus agalactiae percent susceptibility* to antibiotics in 13 Lebanese hospitals during 2015/2016 a. (DOCX 103 kb) [file 13756_2019_487_MOESM7_ESM.docx]

**Additional file 7**

**Table 1.** *Streptococcus pneumoniae, Streptococcus viridans, Streptococcus pyogenes*, and *Streptococcus agalactiae* percent susceptibility* to antibiotics in 13 Lebanese hospitals during 2015/2016 ^a^

| **Antibiotics** | ***S. pneumoniae*** | | ***S. viridans*** | | ***S. pyogenes*** | | ***S. agalactiae*** | |
| --- | --- | --- | --- | --- | --- | --- | --- | --- |
|  | No. of tested isolates | % S (Range) | No. of tested isolates | % S (Range) | No. of tested isolates | % S (Range) | No. of tested isolates | % S (Range) |
| Ceftriaxone | NR | NR | 253 | 89 (85-90) | NR | NR | NR | NR |
| Clindamycin | 584 | 75 (67-95) | 332 | 75 (73-100) | 718 | 95 (90-100) | 102 | 77 (64-100) |
| Erythromycin | 584 | 65 (58-90) | 332 | 64 (61-100) | 718 | 93 (89-100) | 102 | 72 (56-100) |
| Levofloxacin | 584 | 99 (98-100) | NR | NR | NR | NR | NR | NR |
| Penicillin^b^ | 562 | 75 (60-79) | 113 | 66 (55-100) | 718 | 100 (100) | 102 | 100 (97-100) |
| Tetracycline | 179 | 74 (69-83) | NR | NR | NR | NR | NR | NR |
| Trimethoprim/sulfamethoxazole | 398 | 55 (32-72) | NR | NR | NR | NR | NR | NR |
| Vancomycin | NR | NR | NR | NR | 718 | 100 (100) | 102 | 100 (100) |

**Key=** NR: not reported, S: Susceptibility, %: Percentage.

**N.B.**

^a^ *S. pneumoniae,* *S. viridans*, *Streptococcus pyogenes*, and *Streptococcus agalactiae* susceptibilities were obtained from 8, 4, 9, and 10 Lebanese hospitals respectively.

^b^ In case of *S. pneumoniae,* penicillin susceptibility results were calculated using data from 3 laboratories, which abided by CLSI and EUCAST guidelines recommendations.

*Susceptibility is represented as mean (%) for each antibiotic-microbe combination and the range is the upper and lower limits of individual % susceptibility from participating centres.
