# Supplementary material for: A compilation of antimicrobial susceptibility data from a network of 13 Lebanese hospitals reflecting the national situation during 2015–2016
Source: Antimicrob Resist Infect Control. 2019 Feb 20;8:41. doi: 10.1186/s13756-019-0487-5 (PMC6381724; doi:10.1186/s13756-019-0487-5)
Supplement: Supplementary file 8 — Table S1. E. coli percent susceptibility to third-generation cephalosporins in countries of the European Union, based on the 2015 and 2016 annual reports of the European Antimicrobial Resistance Surveillance Network (EARS-Net)1,2, and comparison to 2015–2016 Lebanese data. (DOCX 110 kb) [file 13756_2019_487_MOESM8_ESM.docx]

**Additional file 8**

**Table 1.** *E. coli* percent susceptibility to third-generation cephalosporins in countries of the European Union, based on the 2015 and 2016 annual reports of the European Antimicrobial Resistance Surveillance Network (EARS-Net)^1,2^, and comparison to 2015-2016 Lebanese data

| **Country** | **Number of tested isolates** | **Percent susceptibility** | **Odds ratio** | **95% confidence interval** | | **Adjusted p-value** |
| --- | --- | --- | --- | --- | --- | --- |
| **Austria** | 10167 | 90,2 | 0,18 | 0,17 | 0,20 | < 0.001 |
| **Belgium** | 6330 | 89,9 | 0,19 | 0,18 | 0,21 | < 0.001 |
| **Bulgaria** | 443 | 60,0 | 0,93 | 0,75 | 0,98 | 1 |
| **Croatia** | 2091 | 86,4 | 0,27 | 0,23 | 0,30 | < 0.001 |
| **Cyprus** | 272 | 70,7 | 0,70 | 0,54 | 0,91 | 1 |
| **Czech Republic** | 6233 | 85,2 | 0,29 | 0,27 | 0,31 | < 0.001 |
| **Denmark** | 9220 | 93,0 | 0,13 | 0,12 | 0,14 | < 0.001 |
| **Estonia** | 947 | 89,8 | 0,18 | 0,14 | 0,22 | < 0.001 |
| **Finland** | 9084 | 93,5 | 0,12 | 0,11 | 0,13 | < 0.001 |
| **France** | 22364 | 88,9 | 0,21 | 0,20 | 0,22 | < 0.001 |
| **Germany** | 24501 | 89,1 | 0,21 | 0,20 | 0,22 | < 0.001 |
| **Greece** | 2519 | 81,3 | 0,39 | 0,35 | 0,43 | < 0.001 |
| **Hungary** | 4019 | 83,3 | 0,34 | 0,31 | 0,37 | < 0.001 |
| **Iceland** | 365 | 97,1 | 0,05 | 0,03 | 0,09 | < 0.001 |
| **Ireland** | 5623 | 88,6 | 0,22 | 0,20 | 0,24 | < 0.001 |
| **Italy** | 11530 | 70,1 | 0,72 | 0,69 | 0,75 | < 0.001 |
| **Latvia** | 454 | 79,0 | 0,46 | 0,36 | 0,57 | < 0.001 |
| **Lithuania** | 1376 | 84,7 | 0,30 | 0,26 | 0,35 | < 0.001 |
| **Luxembourg** | 765 | 86,9 | 0,26 | 0,21 | 0,31 | < 0.001 |
| **Malta** | 586 | 87,1 | 0,26 | 0,20 | 0,32 | < 0.001 |
| **Netherlands** | 11775 | 94,0 | 0,11 | 0,10 | 0,12 | < 0.001 |
| **Norway** | 6918 | 94,2 | 0,10 | 0,09 | 0,11 | < 0.001 |
| **Poland** | 4329 | 87,2 | 0,25 | 0,23 | 0,28 | < 0.001 |
| **Portugal** | 11160 | 83,9 | 0,32 | 0,31 | 0,34 | < 0.001 |
| **Romania** | 787 | 74,9 | 0,56 | 0,48 | 0,66 | < 0.001 |
| **Slovakia** | 1717 | 70,2 | 0,72 | 0,65 | 0,80 | < 0.001 |
| **Slovenia** | 2746 | 86,9 | 0,25 | 0,23 | 0,28 | < 0.001 |
| **Spain** | 13224 | 86,7 | 0,26 | 0,25 | 0,27 | < 0.001 |
| **Sweden** | 12953 | 92,8 | 0,13 | 0,13 | 0,14 | < 0.001 |
| **United Kingdom** | 27015 | 100 | 0,18 | 0,17 | 0,19 | < 0.001 |
| **Lebanon** | **41816** | **58** | **-** | **-** | **-** | **-** |

References

1. European Centre for Disease Prevention and Control. Antimicrobial resistance surveillance in Europe 2015. Annual Report of the European Antimicrobial Resistance Surveillance Network (EARS-Net). Stockholm: ECDC; 2016.
2. European Centre for Disease Prevention and Control. Antimicrobial resistance surveillance in Europe 2016. Annual Report of the European Antimicrobial Resistance Surveillance Network (EARS-Net). Stockholm: ECDC; 2017.
