# Supplementary material for: A compilation of antimicrobial susceptibility data from a network of 13 Lebanese hospitals reflecting the national situation during 2015–2016
Source: Antimicrob Resist Infect Control. 2019 Feb 20;8:41. doi: 10.1186/s13756-019-0487-5 (PMC6381724; doi:10.1186/s13756-019-0487-5)
Supplement: Supplementary file 9 — Table S1. E. coli percent susceptibility to carbapenems in countries of the European Union, based on the 2015 and 2016 annual reports of the European Antimicrobial Resistance Surveillance Network (EARS-Net)1,2, and comparison to 2015–2016 Lebanese data. (DOCX 113 kb) [file 13756_2019_487_MOESM9_ESM.docx]

**Additional file 9**

**Table 1.** *E. coli* percent susceptibility to carbapenems in countries of the European Union, based on the 2015 and 2016 annual reports of the European Antimicrobial Resistance Surveillance Network (EARS-Net)^1,2^, and comparison to 2015-2016 Lebanese data

| **Country** | **Number of tested isolates** | **Percent susceptibility** | **Odds ratio** | **95% confidence interval** | | **Adjusted p-value** |
| --- | --- | --- | --- | --- | --- | --- |
| **Austria** | 9894 | 99 | 0,004 | 0 | 0,018 | < 0.001 |
| **Belgium** | 6433 | 99,95 | 0,023 | 0,007 | 0,053 | < 0.001 |
| **Bulgaria** | 406 | 99,55 | 0,189 | 0,029 | 0,582 | 0,775 |
| **Croatia** | 2091 | 100 | 0,019 | 0,001 | 0,084 | < 0.001 |
| **Cyprus** | 272 | 100 | 0,149 | 0,007 | 0,648 | 1 |
| **Czech Republic** | 2954 | 100 | 0,014 | 0,001 | 0,059 | < 0.001 |
| **Denmark** | 8717 | 99 | 0,005 | 0 | 0,02 | < 0.001 |
| **Estonia** | 821 | 100 | 0,049 | 0,002 | 0,213 | < 0.001 |
| **Finland** | 9257 | 99 | 0,004 | 0 | 0,019 | < 0.001 |
| **France** | 21410 | 99 | 0,002 | 0 | 0,008 | < 0.001 |
| **Germany** | 24508 | 99 | 0,002 | 0 | 0,007 | < 0.001 |
| **Greece** | 2518 | 98,95 | 0,385 | 0,257 | 0,553 | < 0.001 |
| **Hungary** | 3827 | 100 | 0,011 | 0 | 0,046 | < 0.001 |
| **Iceland** | 354 | 100 | 0,115 | 0,005 | 0,497 | 0,465 |
| **Ireland** | 5604 | 99 | 0,007 | 0 | 0,031 | < 0.001 |
| **Italy** | 11698 | 99,75 | 0,088 | 0,06 | 0,125 | < 0.001 |
| **Latvia** | 438 | 100 | 0,093 | 0,004 | 0,401 | 0,155 |
| **Lithuania** | 1372 | 100 | 0,03 | 0,001 | 0,127 | < 0.001 |
| **Luxembourg** | 765 | 100 | 0,053 | 0,002 | 0,229 | < 0.001 |
| **Malta** | 586 | 100 | 0,069 | 0,003 | 0,299 | < 0.001 |
| **Netherlands** | 11769 | 99 | 0,003 | 0 | 0,015 | < 0.001 |
| **Norway** | 6913 | 99 | 0,006 | 0 | 0,025 | < 0.001 |
| **Poland** | 4052 | 99 | 0,01 | 0 | 0,043 | < 0.001 |
| **Portugal** | 11114 | 99 | 0,004 | 0 | 0,016 | < 0.001 |
| **Romania** | 779 | 98,55 | 0,514 | 0,265 | 0,886 | 1 |
| **Slovakia** | 1581 | 100 | 0,026 | 0,001 | 0,111 | < 0.001 |
| **Slovenia** | 2746 | 100 | 0,015 | 0,001 | 0,064 | < 0.001 |
| **Spain** | 13189 | 99 | 0,003 | 0 | 0,013 | < 0.001 |
| **Sweden** | 12234 | 99,9 | 0,035 | 0,019 | 0,059 | < 0.001 |
| **United Kingdom** | 28259 | 99 | 0,001 | 0 | 0,006 | < 0.001 |
| **Lebanon** | **41813** | **97** | **-** | **-** | **-** | **-** |

References

1. European Centre for Disease Prevention and Control. Antimicrobial resistance surveillance in Europe 2015. Annual Report of the European Antimicrobial Resistance Surveillance Network (EARS-Net). Stockholm: ECDC; 2016.
2. European Centre for Disease Prevention and Control. Antimicrobial resistance surveillance in Europe 2016. Annual Report of the European Antimicrobial Resistance Surveillance Network (EARS-Net). Stockholm: ECDC; 2017.
