# Supplementary material for: A compilation of antimicrobial susceptibility data from a network of 13 Lebanese hospitals reflecting the national situation during 2015–2016
Source: Antimicrob Resist Infect Control. 2019 Feb 20;8:41. doi: 10.1186/s13756-019-0487-5 (PMC6381724; doi:10.1186/s13756-019-0487-5)
Supplement: Supplementary file 10 — Table S1. K. pneumoniae percent susceptibility to third-generation in countries of the European Union, based on the 2015 and 2016 annual reports of the European Antimicrobial Resistance Surveillance Network (EARS-Net)1,2, and comparison to 2015–2016 Lebanese data. (DOCX 110 kb) [file 13756_2019_487_MOESM10_ESM.docx]

**Additional file 10**

**Table 1.** *K. pneumoniae* percent susceptibility to third-generation in countries of the European Union, based on the 2015 and 2016 annual reports of the European Antimicrobial Resistance Surveillance Network (EARS-Net)^1,2^, and comparison to 2015-2016 Lebanese data

| **Country** | **Number of tested isolates** | **Percent susceptibility** | **Odds ratio** | **95% confidence interval** | | **Adjusted p-value** |
| --- | --- | --- | --- | --- | --- | --- |
| **Austria** | 2295 | 91,00 | 0,20 | 0,17 | 0,23 | < 0.001 |
| **Belgium** | 1075 | 78,70 | 0,55 | 0,47 | 0,63 | < 0.001 |
| **Bulgaria** | 256 | 26,25 | 5,47 | 4,16 | 7,27 | < 0.001 |
| **Croatia** | 701 | 52,30 | 1,80 | 1,55 | 2,09 | < 0.001 |
| **Cyprus** | 137 | 62,90 | 1,14 | 0,80 | 1,61 | 1 |
| **Czech Republic** | 2801 | 47,05 | 2,23 | 2,07 | 2,41 | < 0.001 |
| **Denmark** | 2047 | 92,35 | 0,16 | 0,14 | 0,19 | < 0.001 |
| **Estonia** | 276 | 71,75 | 0,84 | 0,64 | 1,08 | 1 |
| **Finland** | 1404 | 96,45 | 0,07 | 0,06 | 0,10 | < 0.001 |
| **France** | 4935 | 70,30 | 0,84 | 0,78 | 0,89 | < 0.001 |
| **Germany** | 4330 | 88,10 | 0,28 | 0,26 | 0,31 | < 0.001 |
| **Greece** | 2366 | 29,00 | 4,85 | 4,43 | 5,32 | < 0.001 |
| **Hungary** | 1426 | 62,65 | 1,18 | 1,06 | 1,32 | 0,372 |
| **Iceland** | 61 | 100,00 | 0,04 | 0,002 | 0,17 | < 0.001 |
| **Ireland** | 839 | 85,90 | 0,33 | 0,27 | 0,39 | < 0.001 |
| **Italy** | 4245 | 44,15 | 2,50 | 2,35 | 2,67 | < 0.001 |
| **Latvia** | 210 | 52,80 | 1,77 | 1,35 | 2,32 | < 0.001 |
| **Lithuania** | 504 | 45,80 | 2,42 | 2,03 | 2,89 | < 0.001 |
| **Luxembourg** | 138 | 67,90 | 0,96 | 0,67 | 1,36 | 1 |
| **Malta** | 194 | 81,05 | 0,47 | 0,32 | 0,66 | < 0.001 |
| **Netherlands** | 2042 | 90,55 | 0,21 | 0,18 | 0,24 | < 0.001 |
| **Norway** | 1512 | 94,60 | 0,11 | 0,09 | 0,14 | < 0.001 |
| **Poland** | 1818 | 35,70 | 3,57 | 3,24 | 3,94 | < 0.001 |
| **Portugal** | 4443 | 56,45 | 1,54 | 1,45 | 1,64 | < 0.001 |
| **Romania** | 614 | 30,65 | 4,45 | 3,75 | 5,30 | < 0.001 |
| **Slovakia** | 934 | 35,75 | 3,56 | 3,11 | 4,08 | < 0.001 |
| **Slovenia** | 504 | 77,20 | 0,59 | 0,47 | 0,72 | < 0.001 |
| **Spain** | 3169 | 78,70 | 0,54 | 0,49 | 0,59 | < 0.001 |
| **Sweden** | 2538 | 95,90 | 0,09 | 0,07 | 0,11 | < 0.001 |
| **United Kingdom** | 4830 | 90,30 | 0,20 | 0,18 | 0,22 | < 0.001 |
| **Lebanon** | **9498** | **63,00** | - | - | - | - |

References

1. European Centre for Disease Prevention and Control. Antimicrobial resistance surveillance in Europe 2015. Annual Report of the European Antimicrobial Resistance Surveillance Network (EARS-Net). Stockholm: ECDC; 2016.
2. European Centre for Disease Prevention and Control. Antimicrobial resistance surveillance in Europe 2016. Annual Report of the European Antimicrobial Resistance Surveillance Network (EARS-Net). Stockholm: ECDC; 2017.
