# Supplementary material for: A compilation of antimicrobial susceptibility data from a network of 13 Lebanese hospitals reflecting the national situation during 2015–2016
Source: Antimicrob Resist Infect Control. 2019 Feb 20;8:41. doi: 10.1186/s13756-019-0487-5 (PMC6381724; doi:10.1186/s13756-019-0487-5)
Supplement: Supplementary file 11 — Table S1. K. pneumoniae percent susceptibility to carbapenems in countries of the European Union, based on the 2015 and 2016 annual reports of the European Antimicrobial Resistance Surveillance Network (EARS-Net)1,2, and comparison to 2015–2016 Lebanese data. (DOCX 106 kb) [file 13756_2019_487_MOESM11_ESM.docx]

**Additional file 11**

**Table 1.** *K. pneumoniae* percent susceptibility to carbapenems in countries of the European Union, based on the 2015 and 2016 annual reports of the European Antimicrobial Resistance Surveillance Network (EARS-Net)^1,2^, and comparison to 2015-2016 Lebanese data

| **Country** | **Number of tested isolates** | **Percent susceptibility** | **Odds ratio** | **95% confidence interval** | | **Adjusted p-value** |
| --- | --- | --- | --- | --- | --- | --- |
| **Austria** | 2220 | 95,85 | 1,01 | 0,96 | 1,20 | 1 |
| **Belgium** | 1058 | 98,30 | 0,37 | 0,22 | 0,57 | < 0.001 |
| **Bulgaria** | 254 | 96,05 | 0,87 | 0,43 | 1,56 | 1 |
| **Croatia** | 703 | 98,69 | 0,28 | 0,13 | 0,50 | < 0.001 |
| **Cyprus** | 137 | 88,30 | 2,79 | 1,58 | 4,59 | < 0.001 |
| **Czech Republic** | 2196 | 99,85 | 0,03 | 0,01 | 0,08 | < 0.001 |
| **Denmark** | 1965 | 99,83 | 0,03 | 0,01 | 0,09 | < 0.001 |
| **Estonia** | 224 | 100 | 0,11 | 0,01 | 0,47 | 0,248 |
| **Finland** | 1428 | 99,84 | 0,03 | 0,01 | 0,10 | < 0.001 |
| **France** | 4772 | 99,55 | 0,09 | 0,06 | 0,14 | < 0.001 |
| **Germany** | 4332 | 99,64 | 0,08 | 0,05 | 0,12 | < 0.001 |
| **Greece** | 2365 | 35,61 | 37,81 | 33,83 | 42,35 | < 0.001 |
| **Hungary** | 1390 | 99,75 | 0,06 | 0,02 | 0,15 | < 0.001 |
| **Iceland** | 56 | 100 | 0,43 | 0,02 | 1,91 | 1 |
| **Ireland** | 842 | 99,39 | 0,13 | 0,05 | 0,28 | < 0.001 |
| **Italy** | 4306 | 98,49 | 0,96 | 0,81 | 1,12 | 1 |
| **Latvia** | 202 | 99,02 | 0,23 | 0,04 | 0,70 | 1 |
| **Lithuania** | 502 | 100 | 0,05 | 0,002 | 0,21 | < 0.001 |
| **Luxembourg** | 138 | 100 | 0,17 | 0,01 | 0,76 | 1 |
| **Malta** | 190 | 66,24 | 1,28 | 0,65 | 2,24 | 1 |
| **Netherlands** | 2038 | 99,90 | 0,02 | 0 | 0,07 | < 0.001 |
| **Norway** | 1510 | 99,95 | 0,02 | 0 | 0,07 | < 0.001 |
| **Poland** | 1783 | 98,49 | 0,32 | 0,21 | 0,47 | < 0.001 |
| **Portugal** | 4425 | 95,65 | 1,01 | 0,95 | 1,15 | 1 |
| **Romania** | 605 | 71,60 | 8,31 | 6,85 | 10,05 | < 0.001 |
| **Slovakia** | 871 | 98,30 | 0,37 | 0,21 | 0,60 | < 0.001 |
| **Slovenia** | 504 | 99,39 | 0,13 | 0,03 | 0,34 | < 0.001 |
| **Spain** | 3160 | 97,85 | 0,46 | 0,36 | 0,59 | < 0.001 |
| **Sweden** | 2431 | 99,94 | 0,02 | 0,003 | 0,06 | < 0.001 |
| **United Kingdom** | 5030 | 99,68 | 0,07 | 0,04 | 0,11 | < 0.001 |
| **Lebanon** | **9498** | **96** | **-** | **-** | **-** | **-** |

References

1. European Centre for Disease Prevention and Control. Antimicrobial resistance surveillance in Europe 2015. Annual Report of the European Antimicrobial Resistance Surveillance Network (EARS-Net). Stockholm: ECDC; 2016.
2. European Centre for Disease Prevention and Control. Antimicrobial resistance surveillance in Europe 2016. Annual Report of the European Antimicrobial Resistance Surveillance Network (EARS-Net). Stockholm: ECDC; 2017.
