# Supplementary material for: A compilation of antimicrobial susceptibility data from a network of 13 Lebanese hospitals reflecting the national situation during 2015–2016
Source: Antimicrob Resist Infect Control. 2019 Feb 20;8:41. doi: 10.1186/s13756-019-0487-5 (PMC6381724; doi:10.1186/s13756-019-0487-5)
Supplement: Supplementary file 12 — Table S1. P. aeruginosa percent susceptibility to carbapenems in countries of the European Union, based on the 2015 and 2016 annual reports of the European Antimicrobial Resistance Surveillance Network (EARS-Net)1,2, and comparison to 2015–2016 Lebanese data. (DOCX 114 kb) [file 13756_2019_487_MOESM12_ESM.docx]

**Additional file 12**

**Table 1.** *P. aeruginosa* percent susceptibility to carbapenems in countries of the European Union, based on the 2015 and 2016 annual reports of the European Antimicrobial Resistance Surveillance Network (EARS-Net)^1,2^, and comparison to 2015-2016 Lebanese data

| **Country** | **Number of tested isolates** | **Percent susceptibility** | **Odds ratio** | **95% confidence interval** | | **Adjusted p-value** |
| --- | --- | --- | --- | --- | --- | --- |
| **Austria** | 1376 | 87,45 | 0,33 | 0,28 | 0,39 | < 0.001 |
| **Belgium** | 621 | 93,25 | 0,18 | 0,13 | 0,24 | < 0.001 |
| **Bulgaria** | 111 | 72,05 | 0,90 | 0,58 | 1,35 | 1 |
| **Croatia** | 517 | 59,60 | 1,56 | 1,31 | 1,87 | < 0.001 |
| **Cyprus** | 107 | 80,15 | 0,57 | 0,34 | 0,90 | 1 |
| **Czech Republic** | 928 | 90,30 | 0,25 | 0,20 | 0,31 | < 0.001 |
| **Denmark** | 895 | 96,50 | 0,08 | 0,06 | 0,12 | < 0.001 |
| **Estonia** | 70 | 83,55 | 0,53 | 0,28 | 0,94 | 1 |
| **Finland** | 693 | 94,65 | 0,13 | 0,09 | 0,18 | < 0.001 |
| **France** | 3893 | 84,00 | 0,44 | 0,40 | 0,48 | < 0.001 |
| **Germany** | 2260 | 85,00 | 0,41 | 0,36 | 0,46 | < 0.001 |
| **Greece** | 1374 | 58,75 | 1,62 | 1,44 | 1,82 | < 0.001 |
| **Hungary** | 1509 | 65,45 | 1,22 | 1,09 | 1,36 | 0,155 |
| **Iceland** | 29 | 97,05 | 0,09 | 0,004 | 0,44 | 0,31 |
| **Ireland** | 438 | 92,30 | 0,19 | 0,13 | 0,27 | < 0.001 |
| **Italy** | 2288 | 76,75 | 0,70 | 0,63 | 0,78 | < 0.001 |
| **Latvia** | 29 | 76,65 | 0,75 | 0,29 | 1,67 | 1 |
| **Lithuania** | 115 | 78,50 | 0,58 | 0,36 | 0,90 | 1 |
| **Luxembourg** | 55 | 92,60 | 0,19 | 0,06 | 0,46 | < 0.001 |
| **Malta** | 65 | 85,75 | 0,38 | 0,17 | 0,72 | 0,62 |
| **Netherlands** | 1043 | 96,15 | 0,09 | 0,07 | 0,13 | < 0.001 |
| **Norway** | 453 | 93,80 | 0,15 | 0,10 | 0,22 | < 0.001 |
| **Poland** | 651 | 68,40 | 1,01 | 0,85 | 1,19 | 1 |
| **Portugal** | 2418 | 80,50 | 0,56 | 0,50 | 0,62 | < 0.001 |
| **Romania** | 185 | 41,05 | 3,30 | 2,46 | 4,45 | < 0.001 |
| **Slovakia** | 444 | 52,90 | 2,13 | 1,76 | 2,57 | < 0.001 |
| **Slovenia** | 284 | 82,40 | 0,49 | 0,36 | 0,67 | < 0.001 |
| **Spain** | 1714 | 77,95 | 0,65 | 0,58 | 0,74 | < 0.001 |
| **Sweden** | 870 | 91,25 | 0,23 | 0,18 | 0,29 | < 0.001 |
| **United Kingdom** | 2607 | 96,25 | 0,11 | 0,09 | 0,13 | < 0.001 |
| **Lebanon** | 9005 | **70** | - | - | - | - |

References

1. European Centre for Disease Prevention and Control. Antimicrobial resistance surveillance in Europe 2015. Annual Report of the European Antimicrobial Resistance Surveillance Network (EARS-Net). Stockholm: ECDC; 2016.
2. European Centre for Disease Prevention and Control. Antimicrobial resistance surveillance in Europe 2016. Annual Report of the European Antimicrobial Resistance Surveillance Network (EARS-Net). Stockholm: ECDC; 2017.
