# Supplementary material for: A compilation of antimicrobial susceptibility data from a network of 13 Lebanese hospitals reflecting the national situation during 2015–2016
Source: Antimicrob Resist Infect Control. 2019 Feb 20;8:41. doi: 10.1186/s13756-019-0487-5 (PMC6381724; doi:10.1186/s13756-019-0487-5)
Supplement: Supplementary file 13 — Table S1. Acinetobacter spp. percent susceptibility to carbapenems in countries of the European Union, based on the 2015 and 2016 annual reports of the European Antimicrobial Resistance Surveillance Network (EARS-Net)1,2, and comparison to 2015–2016 Lebanese data. (DOCX 112 kb) [file 13756_2019_487_MOESM13_ESM.docx]

**Additional file 13**

**Table 1.** *Acinetobacter* spp percent susceptibility to carbapenems in countries of the European Union, based on the 2015 and 2016 annual reports of the European Antimicrobial Resistance Surveillance Network (EARS-Net)^1,2^, and comparison to 2015-2016 Lebanese data

| **Country** | **Number of tested isolates** | **Percent susceptibility** | **Odds ratio** | **95% confidence interval** | | **Adjusted p-value** |
| --- | --- | --- | --- | --- | --- | --- |
| **Austria** | 145 | 89,15 | 0,019 | 0,011 | 0,031 | < 0.001 |
| **Belgium** | 102 | 98,70 | 0,003 | 0,001 | 0,01 | < 0.001 |
| **Bulgaria** | 233 | 25,70 | 0,438 | 0,324 | 0,6 | < 0.001 |
| **Croatia** | 381 | 8,25 | 1,652 | 1,155 | 2,446 | 0,567 |
| **Cyprus** | 87 | 22,75 | 0,58 | 0,349 | 1,013 | 1 |
| **Czech Republic** | 117 | 95,75 | 0,007 | 0,002 | 0,016 | < 0.001 |
| **Denmark** | 134 | 97,70 | 0,004 | 0,001 | 0,01 | < 0.001 |
| **Finland** | 71 | 98,85 | 0,002 | 0 | 0,011 | < 0.001 |
| **France** | 878 | 93,65 | 0,01 | 0,008 | 0,014 | < 0.001 |
| **Germany** | 763 | 94,25 | 0,009 | 0,007 | 0,012 | < 0.001 |
| **Greece** | 1844 | 5,55 | 2,542 | 2,052 | 3,178 | < 0.001 |
| **Hungary** | 868 | 43,10 | 0,2 | 0,17 | 0,236 | < 0.001 |
| **Ireland** | 149 | 13,00 | 1.01 | 0.905 | 1.10 | 1 |
| **Italy** | 1366 | 21,60 | 0,552 | 0,472 | 0,648 | < 0.001 |
| **Latvia** | 143 | 28,95 | 0,378 | 0,262 | 0,555 | < 0.001 |
| **Lithuania** | 160 | 18,80 | 0,657 | 0,443 | 1,005 | 1 |
| **Netherlands** | 177 | 97,95 | 0,003 | 0,001 | 0,007 | < 0.001 |
| **Norway** | 65 | 95,30 | 0,008 | 0,002 | 0,021 | < 0.001 |
| **Poland** | 635 | 34,20 | 0,293 | 0,243 | 0,354 | < 0.001 |
| **Portugal** | 513 | 45,20 | 0,189 | 0,155 | 0,23 | < 0.001 |
| **Romania** | 349 | 16,75 | 0,746 | 0,56 | 1,011 | 1 |
| **Slovakia** | 251 | 71,70 | 0,06 | 0,045 | 0,08 | < 0.001 |
| **Slovenia** | 91 | 59,00 | 0,109 | 0,071 | 0,167 | < 0.001 |
| **Spain** | 201 | 42,00 | 0,212 | 0,158 | 0,285 | < 0.001 |
| **Sweden** | 118 | 97,95 | 0,003 | 0 | 0,009 | < 0.001 |
| **United Kingdom** | 716 | 98,85 | 0,002 | 0,001 | 0,004 | < 0.001 |
| **Lebanon** | 3900 | **13** | - | - | - | - |

References

1. European Centre for Disease Prevention and Control. Antimicrobial resistance surveillance in Europe 2015. Annual Report of the European Antimicrobial Resistance Surveillance Network (EARS-Net). Stockholm: ECDC; 2016.
2. European Centre for Disease Prevention and Control. Antimicrobial resistance surveillance in Europe 2016. Annual Report of the European Antimicrobial Resistance Surveillance Network (EARS-Net). Stockholm: ECDC; 2017.
