# Supplementary material for: A compilation of antimicrobial susceptibility data from a network of 13 Lebanese hospitals reflecting the national situation during 2015–2016
Source: Antimicrob Resist Infect Control. 2019 Feb 20;8:41. doi: 10.1186/s13756-019-0487-5 (PMC6381724; doi:10.1186/s13756-019-0487-5)
Supplement: Supplementary file 15 — Table S1. S. pneumonaie percent susceptibility to penicillin countries of the European Union during 2015 and 2016, based on the 2015 and 2016 annual reports of the European Antimicrobial Resistance Surveillance Network (EARS-Net)1,2, and comparison to 2015–2016 Lebanese data. (DOCX 110 kb) [file 13756_2019_487_MOESM15_ESM.docx]

**Additional file 15**

**Table 1.** *S. pneumonaie* percent susceptibility to penicillin countries of the European Union during 2015 and 2016, based on the 2015 and 2016 annual reports of the European Antimicrobial Resistance Surveillance Network (EARS-Net)^1,2^, and comparison to 2015-2016 Lebanese data

| **Country** | **Number of tested isolates** | **Percent susceptibility** | **Odds ratio** | **95% confidence interval** | | **Adjusted p-value** |
| --- | --- | --- | --- | --- | --- | --- |
| **Austria** | 884 | 95,50 | 0,14 | 0,10 | 0,21 | < 0.001 |
| **Belgium** | 2689 | 99,50 | 0,02 | 0,01 | 0,03 | < 0.001 |
| **Bulgaria** | 68 | 74,90 | 1,01 | 0,55 | 1,78 | 1 |
| **Croatia** | 281 | 79,55 | 0,79 | 0,55 | 1,11 | 1 |
| **Cyprus** | 17 | 100 | 0,95 | 0,26 | 2,78 | 1 |
| **Czech Republic** | 550 | 96,15 | 0,12 | 0,07 | 0,19 | < 0.001 |
| **Denmark** | 1454 | 94,60 | 0,17 | 0,13 | 0,23 | < 0.001 |
| **Estonia** | 184 | 96,80 | 0,10 | 0,04 | 0,22 | < 0.001 |
| **Finland** | 1383 | 88,50 | 0,39 | 0,30 | 0,51 | < 0.001 |
| **France** | 2114 | 75,90 | 0,96 | 0,77 | 1,19 | 1 |
| **Germany** | 1973 | 94,90 | 0,15 | 0,12 | 0,20 | < 0.001 |
| **Hungary** | 355 | 88,65 | 0,38 | 0,26 | 0,56 | < 0.001 |
| **Iceland** | 44 | 82,75 | 0,68 | 0,29 | 1,43 | 1 |
| **Ireland** | 666 | 83,00 | 0,62 | 0,47 | 0,81 | 0,06 |
| **Italy** | 788 | 90,60 | 0,31 | 0,23 | 0,42 | < 0.001 |
| **Latvia** | 120 | 90,00 | 0,34 | 0,17 | 0,61 | < 0.001 |
| **Lithuania** | 186 | 83,85 | 0,58 | 0,37 | 0,89 | 0,72 |
| **Luxembourg** | 78 | 91,30 | 0,35 | 0,15 | 0,71 | 0,24 |
| **Malta** | 30 | 77,50 | 0,96 | 0,77 | 1,19 | 1 |
| **Netherlands** | 2554 | 98,00 | 0,06 | 0,05 | 0,09 | < 0.001 |
| **Norway** | 929 | 95,10 | 0,15 | 0,11 | 0,22 | < 0.001 |
| **Poland** | 554 | 78,15 | 0,82 | 0,62 | 1,08 | 1 |
| **Portugal** | 1681 | 88,30 | 0,40 | 0,31 | 0,51 | < 0.001 |
| **Romania** | 97 | 59,95 | 2,03 | 1,29 | 3,17 | 0,12 |
| **Slovakia** | 40 | 85,05 | 0,65 | 0,26 | 1,43 | 1 |
| **Slovenia** | 592 | 92,15 | 0,26 | 0,18 | 0,37 | < 0.001 |
| **Spain** | 1308 | 75,75 | 0,96 | 0,77 | 1,21 | 1 |
| **Sweden** | 1302 | 91,55 | 0,26 | 0,20 | 0,35 | < 0.001 |
| **United Kingdom** | 4296 | 93,65 | 0,18 | 0,14 | 0,23 | < 0.001 |
| **Lebanon** | 562 | **75** | - | - | - | - |

References

1. European Centre for Disease Prevention and Control. Antimicrobial resistance surveillance in Europe 2015. Annual Report of the European Antimicrobial Resistance Surveillance Network (EARS-Net). Stockholm: ECDC; 2016.
2. European Centre for Disease Prevention and Control. Antimicrobial resistance surveillance in Europe 2016. Annual Report of the European Antimicrobial Resistance Surveillance Network (EARS-Net). Stockholm: ECDC; 2017.
